# Synthesis and Antiviral Properties against SARS-CoV-2 of Epoxybenzooxocino[4,3-*b*]Pyridine Derivatives

**DOI:** 10.3390/molecules27123701

**Published:** 2022-06-09

**Authors:** Alena L. Stalinskaya, Nadezhda V. Martynenko, Zarina T. Shulgau, Alexandr V. Shustov, Viktoriya V. Keyer, Ivan V. Kulakov

**Affiliations:** 1 Institute of Chemistry, Tyumen State University, 15a Perekopskaya St., 625003 Tyumen, Russia; a.l.stalinskaya@utmn.ru (A.L.S.); n.v.martynenko@utmn.ru (N.V.M.); 2 National Center for Biotechnology, 13/5 Kurgalzhynskoe road, Nur-Sultan 010000, Kazakhstan; shulgau@biocenter.kz (Z.T.S.); shustov@biocenter.kz (A.V.S.); keer@biocenter.kz (V.V.K.)

**Keywords:** epoxybenzooxocinopyridine derivatives, antiviral activity, SARS-CoV-2

## Abstract

The COVID-19 pandemic is ongoing as of mid-2022 and requires the development of new therapeutic drugs, because the existing clinically approved drugs are limited. In this work, seven derivatives of epoxybenzooxocinopyridine were synthesized and tested for the ability to inhibit the replication of the SARS-CoV-2 virus in cell cultures. Among the described compounds, six were not able to suppress the SARS-CoV-2 virus’ replication. One compound, which is a derivative of epoxybenzooxocinopyridine with an attached side group of 3,4-dihydroquinoxalin-2-one, demonstrated antiviral activity comparable to that of one pharmaceutical drug. The described compound is a prospective lead substance, because the half-maximal effective concentration is 2.23 μg/μL, which is within a pharmacologically achievable range.

## 1. Introduction

**Figure 1:**
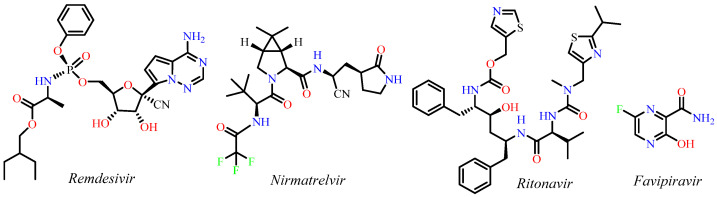
Structures of antiviral drugs against the SARS-CoV-2 virus that have or had clinical approval.

Heterocyclic compounds are an endless source of biologically active compounds and drugs. The novel coronavirus disease 2019 (COVID-19), caused by the severe acute respiratory syndrome virus coronavirus-2 (SARS-CoV-2), has led to a major global emergency [1]. The COVID-19 pandemic is ongoing as of mid-2022, requiring new treatment and prevention tools. Quite a limited number of drugs having direct antiviral action against SARS-CoV-2 are available in practical medicine [2]. The U.S. Food and Drug Administration (FDA) has granted unrestricted clinical approval only to remdesivir [3] (Figure 1). In addition, an Emergency Use Authorization (EUA) was issued for paxlovid, which is a combination of nirmatrelvir and ritonavir [4] (Figure 1). However, recently, the EUA for paxlovid was terminated because paxlovid appeared ineffective against the omicron strain of SARS-CoV-2, which is currently the main circulating strain. Many Old World countries have approved favipiravir [5] (Figure 1), despite the fact that favipiravir has shown little therapeutic benefit in clinical trials and the drug is toxic at therapeutic doses [6]. The mentioned drugs are direct-acting antivirals (DAA) against SARS-CoV-2, i.e., they are capable of specifically recognizing viral proteins, binding to them and inhibiting viral enzymes’ functions. The search for new lead compounds belonging to the DAA class is a practical necessity. With the goal of finding new lead substances, in this study, we screened compounds belonging to the epoxybenzooxocinopyridine class for activity against the SARS-CoV-2 virus. This work continues the research of the authors, which began with the search for the efficient synthesis of compounds built around the epoxybenzo[7,8]oxocino[4,3-*b*]pyridine skeleton [7].

**Scheme 1:**
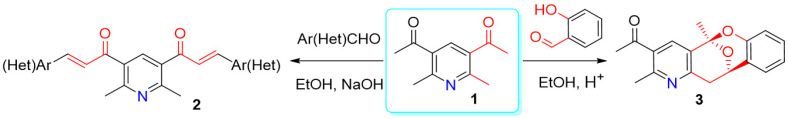
Reaction of pyridine **1** with aryl-aldehydes results in different products, bisazachalcones **2** or epoxybenzo[7,8]oxocino[4,3-*b*]pyridine **3**, as was previously described by the authors in [7,8].

Previously, we have shown that the reaction of 3,5-diacetyl-2,6-dimethylpyridine **1** with aryl-aldehydes under certain conditions does not lead to the formation of symmetrical α,β-unsaturated ketones 2 [8], but proceeds via Claisen–Schmidt condensation and intramolecular cyclization (Scheme 1). For example, the final product of the reaction of compound **1** with salicylaldehyde is epoxybenzo[7,8]oxocino[4,3-*b*]pyridine **3** (Scheme 1).

The resulting epoxybenzooxocinopyridine skeleton in product **3** resembles the structure of the integrastatins epicoccolide A [9] and epicocconigron A [10], which are biologically active natural compounds. Integrastatins exhibit inhibitory properties against human immunodeficiency virus (HIV-1) [11], thus justifying further research in this class of substances to find a remedy against SARS-CoV-2 [12].

We synthesized a variety of derivatives of **3** using 20 different substituted salicylaldehyde synthons in the condensation reaction [7].

## 2. Results

### 2.1. Chemistry

The presence of an acetyl group in **3** makes it possible to carry out chemical modifications and introduce additional pharmacophores while maintaining the integrity of the tetracyclic skeleton.

Since the carbonyl group readily reacts with N-nucleophiles, we have taken this route of addition of aroylhydrazones. The latter were chosen primarily as additional pharmacophores, since aroylhydrazones are widely used in medicine as broad-spectrum bactericides [13,14,15] and anti-tuberculosis drugs [16,17,18]. Other examples of medical applications of these compounds are as anti-inflammatory [19] and antidepressant agents [20].

**Scheme 2:**
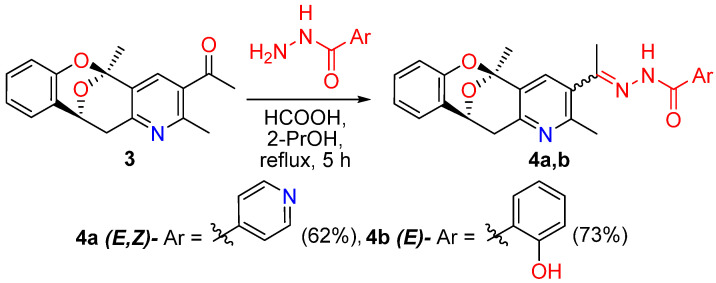
Addition of the aroylhydrazone group to the tetracyclic skeleton of epoxybenzo[7,8]oxocino[4,3-*b*]pyridine.

Aroylhydrazone derivatives **3** were synthesized by condensation with hydrazides of isonicotinic and salicylic acids to give compounds **4a** and **4b** (Scheme 2).

According to ^1^H and ^13^C NMR spectroscopy, reaction product **4a** is a mixture of *E*- and *Z*-isomers present in a ratio of 1:1, as evidenced by the splitting of the proton signals of 5-CH_3_ methyl groups (singlets at 1.90 and 1.93 ppm), 2-CH_3_ (singlets at 2.28 and 2.31 ppm), and 3-CNCH_3_ (singlets at 2.38 and 2.53 ppm) and methylene protons H-12a of the oxocine ring (2 doublets at 3.00, 3.04 ppm).

The results obtained confirm the high reactivity of the acetyl group in compound **3**, which suggests its further use for chemical modifications.

The presence of an acetyl group in compound **3** indicates its possible participation in various aldol-type condensation reactions, including the ester condensation reaction [21,22], in which oxocinopyridine **3** will act as the methylene component. The resulting ester condensation product—for example, α,γ-diketo acid from the corresponding reaction of oxocin **3** with diethyl oxalate—can be an excellent intermediate for further chemical modifications. In addition, α,γ-diketo acids are known to be powerful inhibitors of HIV-1 integrase [23,24,25,26].

**Scheme 3:**
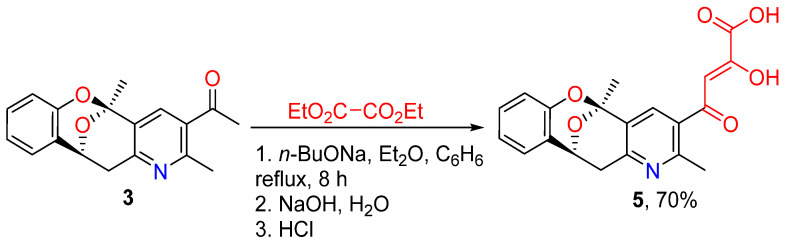
Synthesis of a diketo acid adduct of epoxybenzooxocinopyridine.

We carried out Claisen ester condensation of compound **3** with diethyl oxalate. The reaction was carried out according to the classical method by refluxing in a mixture of solvents Et_2_O–C_6_H_6_ in the presence of *n*-BuONa as a base. As a result, α,γ-diketo acid derivative **5** was obtained with good yield (70%) (Scheme 3).

Structure **5** was confirmed by ^1^H and ^13^C NMR spectroscopy. Compound **5**, dissolved in CDCl_3_, is completely in the enol form, which follows from the data of ^1^H NMR spectroscopy.

It is known that α,γ-diketo acids can be used in cycle-forming reactions with N,N- and N,O-binucleophiles, such as ortho-phenylenediamine, ortho-aminophenol, or 2-amino-3-hydroxypyridine, to produce 3,4-dihydroquinoxalin-2(1*H*)-one and 3,4-dihydro-2*H*-benzo[b][1,4]oxazin-2-one. The two latter compounds are noteworthy because of their pronounced antibacterial, anti-inflammatory, and antitumor activities [27,28,29,30,31,32,33,34].

**Scheme 4:**
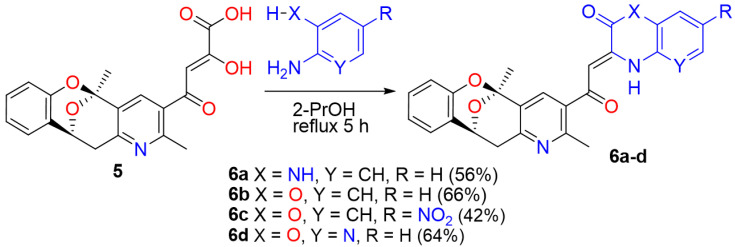
Use of adduct **5** to obtain epoxybenzooxocinopyridine derivatives containing additional pharmacophores dihydroquinoxalin-2-one or dihydrobenzooxazin-2-one.

We carried out the heterocyclization reaction of α,γ-diketo acid **5** with the above binucleophiles (Scheme 4).

Products **6a**–**d** were isolated from the resulting reaction mixture and crystallized to form yellow to orange crystals. ^1^H and ^13^C NMR confirmed the expected structures. The signal of NH protons in compounds **6a**–**d** is in a weak field (at 12–13 ppm), which indicates the formation of an intramolecular hydrogen bond with carbonyl oxygen.

As a result of the carried out chemical modifications, the bicyclic oxocine system turned out to be very stable and did not decompose in either acidic or basic media. The stability of the bicyclic oxocine system is confirmed by the presence of the corresponding signals in the NMR spectra of the synthesized derivatives (in the ^1^H NMR spectra of oxocines, one can observe a characteristic signal of the diastereotopic methylene protons and the methine proton of the epoxybenzooxocine fragment, which are manifested (for example, compound **6b**) as a doublet at 3.07 ppm (^2^*J* = 17.4 Hz), doublet of doublets at 3.66 ppm (^2^*J* = 17.4, ^3^*J* = 5.5 Hz), and a doublet at 5.41 ppm (^3^*J* = 5.5 Hz)) (See Supplementary Materials: S10, ^1^H NMR spectrum).

### 2.2. Biological Activity

Given the importance of discovering new lead substances for SARS-CoV-2 antivirals, compounds **3**, **4a**,**b**, **5**, and **6a**–**c** were tested for antiviral activity in cell cultures. The half-maximal effective concentration (EC_50_) in Vero E6 cultures was determined using a rapid test, as described in the Materials and Methods section. In this rapid screen, virus-infected cells are grown in the wells of a 96-well plate and a compound of interest is added to the growing cells. The range of concentrations is tested simultaneously. One long row of the plate contains one concentration, and the eight long rows differ by the concentration. The test readout is the number of wells per row that exhibit a virus-induced cytopathic effect (CPE). The numbers are processed using the Reed–Muench experimental scheme, e.g., as presented in the work [35] to determine the EC_50_. The EC_50_ is the concentration at which the compound of interest reduces the number of CPE-positive wells by half.

Cytotoxicity is the reduction of cell viability in cultures in the presence of toxic substances. Cytotoxicity also reduces viral replication because viruses need healthy cells to replicate. Cytotoxicity is an undesirable effect for prospective lead substances. In this work, cytotoxicity was measured by determining the 50% inhibitory concentration (IC_50_), which is the concentration that reduces the number of living cells by half. The test is similar to the EC_50_ definition but does not include virus infection. Quantitative live cell density data allow non-linear regression to be used to calculate IC_50_.

Table 1 shows the IC_50_ and EC_50_ values for the seven compounds **3**, **4a**,**b**, **5**, and **6a**–**c** and one pharmaceutical drug with known activity against SARS-CoV-2 that was used as a control for the antiviral action and cytotoxicity.

### 2.3. Cytotoxicity Studies

**Figure 2:**
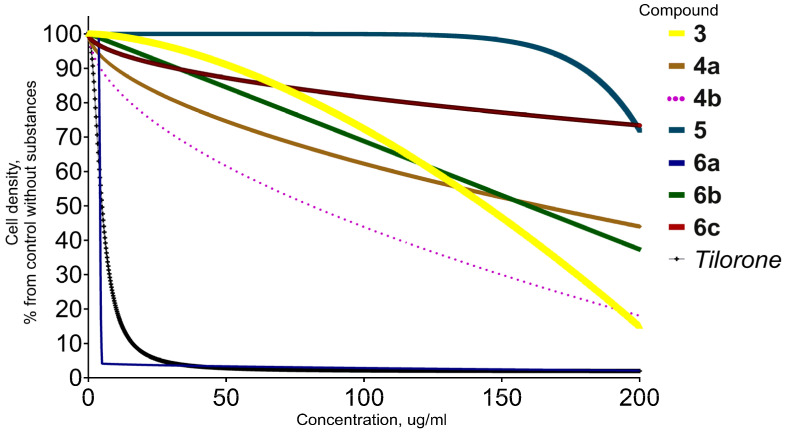
Dependence of cytotoxicity on concentration. The compounds listed in Table 1 were tested to measure cytotoxicity. Tilorone is a pharmaceutical drug known to inhibit the SARS-CoV-2 virus’ replication.

As expected for any substance, there is a concentration above which the substance is toxic to cells. Cytotoxicity manifests itself in slowing down cell divisions, increasing the proportion of dead cells in culture, and, ultimately, in the death of the culture. Cytotoxicity was measured using the MTT test [35], which employs the ability of only living cells to convert the water-soluble compound MTT into water-insoluble formazan. The formazan product can be dissolved in DMSO and measured with a photometer to give an optical density that is proportional to the number of live cells in cultures. Figure 2 shows how the cytotoxicity depends on concentrations of the compounds in this study. Compound **6a** has an IC_50_ of 6.0 µg/mL, which is significantly (at least 33 times) lower than the IC_50_ for other synthesized compounds (Table 1). Since cytotoxicity is also a manifestation of biological activity, such a low concentration means that **6a** affects an unknown cellular function necessary for viability. The pharmaceutical drug tilorone is also toxic, with an IC_50_ of 7.8 µg/mL.

### 2.4. Antiviral Properties

**Figure 3:**
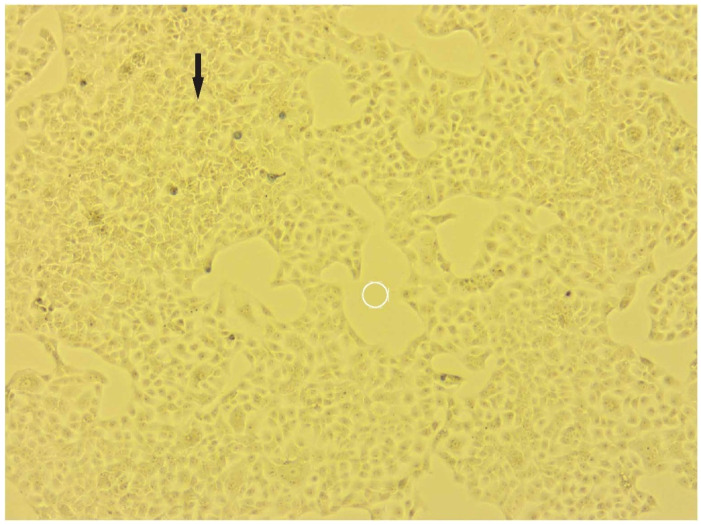
Photograph of a healthy Vero E6 culture, magnification 50×.

**Figure 4:**
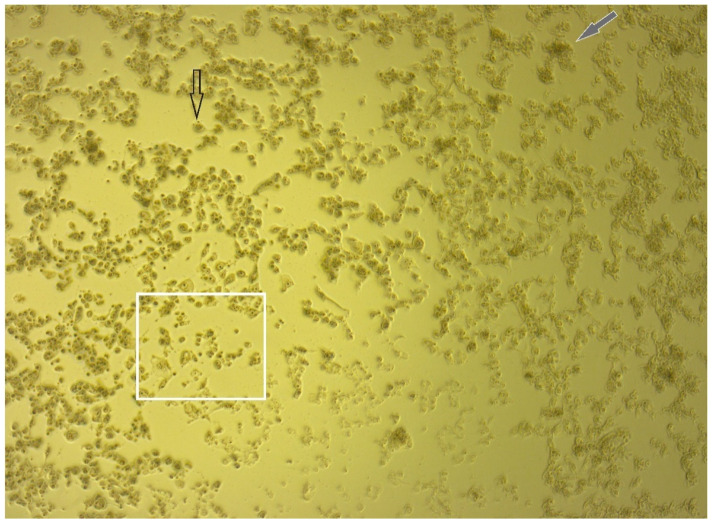
Photograph of an infected Vero E6 culture with typical signs of the virus-induced cytopathic effect, magnification 50×.

Compounds **3**, **4a**,**b**, **5**, and **6a**–**c** were added to cultures of Vero E6 cells and subsequently the cultures were infected with the SARS-CoV-2 virus. The main characteristic of the readout of the test was the presence of a virus-induced CPE, which was represented by pathologic changes in cell morphology. This inhibition of CPE development was found only for **6a**, while **3**, **4a**,**b**, **5**, and **6b**,**c** did not affect the development of CPE at any concentration up to the tested limit of 200 µg/mL. As expected, the pharmaceutical drug tilorone inhibited the SARS-CoV-2 virus-induced CPE, with an EC_50_ at 4.29 µg/mL. For **6a** at 2.47 μg/mL, one in ten wells showed the CPE. Eight of ten wells presented the CPE at a concentration of **6a** 0.82 μg/mL. The data translate to an EC_50_ value for **6a** of 2.23 µg/mL. Figure 3 presents a photograph of a healthy Vero E6 culture, and Figure 4 illustrates this test by showing the appearance of a typical CPE.

Thus, **6a** has prospects for use as a lead substance since the selectivity index (SI) is 2.7 (i.e., the effective virus-inhibiting concentration EC_50_ is 2.7 times lower than the cytotoxic concentration IC_50_). For comparison, tilorone has an SI of 1.8.

Uninfected Vero E6 cells have a typical epithelial cell morphology and have translucent cytoplasm. Normally, there are dead cells in a small amount. Dead cells are small, round, and have dark cytoplasm. In Figure 3, the black arrow points to the confluent monolayer. The white circle shows the area not occupied by cells because the cells did not have enough time to propagate.

Figure 4 shows a microscopic visualization of a virus-induced CPE. The cell density in the presented culture is low because many dead cells have been dislodged from the support. In Figure 4, the arrow with the black outline points to one dead cell. The gray arrow points to a piece of post-cellular debris. The square shape encloses an area that has a typical appearance of dead cells.

As can be seen from the EC_50_ values in Table 1, compound **6a** inhibits the replication of the SARS-CoV-2 virus at a concentration within a pharmacologically achievable range. Compared to other compounds, **6a** is more cytotoxic, indicating higher overall biological activity. Moreover, **6a** has a higher selectivity index than tilorone, which is also toxic but is proposed as an option against SARS-CoV-2 [36,37].

Compound **6a** differs from its closest related structure **6b** only by a different angular heteroatom in the attached pharmacophoric group. However, this difference leads to a significant change in the biological activity, including the antiviral activity for **6a** but not **6b**. As an explanation for why one compound exhibits the antiviral activity and the other with a similar structural skeleton does not, we postulate that **6a** undergoes intracellular metabolic conversion to a pharmacologically active molecule.

### 2.5. Methods and Techniques for Molecular Docking

For a preliminary assessment of the search for a possible biological target for one of the compounds (**6a**), which showed the best activity, molecular docking was carried out. Molecular docking was carried out using the AutoDock Vina software package [38]. The obtained results were visualized using the BIOVIA Discovery Studio 2020 software package [39]. The original structures of protein molecules were taken from the public database of the RCSB Protein Data Bank [40].

The protein structure of SARS-CoV-2 RNA-dependent RNA polymerase (PDB code 7AAP) was chosen as the target protein.

**Figure 5:**
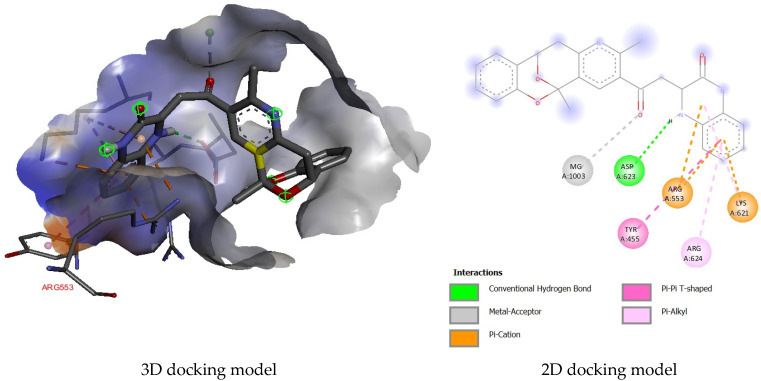
Complex between **6a** and SARS-CoV-2 RNA-dependent RNA polymerase (7AAP).

According to the results of molecular docking, compound **6a** in complex with 7AAP forms one metal–acceptor interaction with magnesium atom 1003, one conventional hydrogen bond with amino acid residue ASP623, two Pi–cation bonds with amino acid residues ARG553 and LYS621, one Pi–alkyl interaction with amino acid residue ARG624, and one Pi–Pi T-shaped interaction with amino acid residue TYR455 (Figure 5).

Comparison of the obtained affinity results for compound **6a** and the reference drug (tilorone) showed that compound **6a** forms many more active interactions with the receptors of the SARS-CoV-2 RNA-dependent RNA polymerase target protein (PDB code 7AAP) (Table 2).

Thus, a protein of the RNA-dependent RNA polymerase of the SARS-CoV-2 virus can serve as a likely biological target for the molecule of compound **6a**.

## 3. Conclusion

Seven compounds were synthesized and tested for virus-inhibiting activity against the SARS-CoV-2 virus. All seven compounds have a common structural framework of epoxybenzooxocinopyridine and differ by the attached side groups, which are: pyridinyl and phenyl groups attached through an ethylidene hydrazide linker; α,γ-diketo acid; dihydroquinoxalin-2-one; dihydrobenzooxazin-2-one; and a nitro derivative of dihydrobenzooxazin-2-one. Among the compounds, six are not significantly cytotoxic and exhibit no ability to suppress the SARS-CoV-2 virus’ replication. One compound, which is epoxybenzooxocinopyridine with the attached dihydroquinoxalin-2-one group, is cytotoxic and also inhibits the SARS-CoV-2 virus’ replication at a concentration within a pharmacologically achievable range. This compound is a promising lead substance for further research on antivirals.

## 4. Materials and Methods

FTIR spectra were obtained with an Agilent Cary 630 spectrophotometer in a thin sample layer on a crystal attachment. ^1^H and ^13^C NMR spectra were recorded on the Bruker DRX400 (400 and 100 MHz, respectively) and Bruker AVANCE 500 (500 and 125 MHz, respectively) instruments, using DMSO-*d_6_* (compound **6d**) or CDCl_3_ (remaining compounds), and the internal standard was TMS or residual solvent signals (7.25 and 77.0 ppm in the case of CDCl_3_ for ^1^H and ^13^C nuclei, respectively; 2.49 and 39.9 ppm ^1^H and for ^13^C nuclei in DMSO-*d_6_*).

Samples were analyzed by HPLC-MS on an Agilent 1260 Infinity II chromatograph coupled to an Agilent 6545 LC/Q-TOF high-resolution mass spectrometer with a Dual AJS ESI ionization source operating in positive ion mode using the following parameters: capillary voltage: 4000 V; spray pressure: 20 (psi); drying gas: 10 L/min; gas temperature: 325 °C; sheathed gas flow: 12 L/min; shielding gas temperature: 400 °C; nozzle voltage: 0 V, fragmentation voltage: 180 V; skimmer voltage: 45 V; octopole RF: 750 V. Mass spectra with LC/MS accuracy were recorded in the range 100–1000 *m*/*z*, scan rate 1.5 spectrum/s.

Chromatographic separation was carried out on ZORBAX RRHD Eclipse Plus C18 (2.1 × 50 mm, particle size 1.8 µm) columns. Column temperature during the analysis was maintained at 35 °C. The mobile phase was formed by eluents A and B. In the positive ionization mode, 0.1% formic acid solution in deionized water was used as eluent A, and 0.1% formic acid solution in acetonitrile was used as eluent B. Chromatographic separation was performed with elution according to the following scheme: 0–10 min 95% A, 10–13 min 100% B, 13–15 min 95% A. The flow of the mobile phase was maintained at 400 μL/min throughout the analysis. In all experiments, the sample injection volume was 1 μL. The sample was prepared by dissolving the entire sample (in 1000 μL) in methanol (for HPLC). Sample dilution was carried out immediately before analysis.

The recorded data were processed using Agilent MassHunter 10.0 software (Agilent Technologies, Santa Clara, CA, USA).

Melting points were determined using a Stuart SMP10 hot bench. Monitoring of the reaction course and the purity of the products was carried out by TLC on Sorbfil plates and visualized using iodine vapor or UV light.

### 4.1. Synthesis of Compounds

1-(2,5-dimethyl-11,12-dihydro-5*H*-5,11-epoxybenzo[7,8]oxocino[4,3-*b*]pyridin-3-yl)ethan-1-one 3 was synthesized according to published procedures [7].

General procedure for the synthesis of *N*-(ethan-1-yl-1-ylidene)benzohydrazides **4a**,**b.** A solution of oxocino [4,3-*b*]pyridine **3** (478 mg, 2.5 mmol) in 2-PrOH was treated with the appropriate hydrazide (5.0 mmol) and 1 drop of formic acid. The reaction mixture was heated at reflux for 5 h. The white precipitate was obtained by filtering, washed with 2-PrOH, and air-dried.

(*E,Z*)-*N*′-(1-(2,5-dimethyl-11,12-dihydro-5*H*-5,11-epoxybenzo[7,8]oxocino[4,3-*b*]pyridin-3-yl)ethylidene)isonicotinohydrazide **4a**. Yield: 631 mg (62%); White crystals, mp. 244–246 °C (2-PrOH). IR (ν_max_, cm^−1^) 3429 (-NH), 1675 (C=O), 1234 (-C-O-C-). ^1^H NMR (400 MHz, CDCl_3_) δ ppm 1.90, 1.93 (2 s, 3H, 5-CH_3_ (*E+Z*)), 2.28, 2.31 (2 s, 3H, 2-CH_3_ (*E+Z*)), 2.38, 2.53 (2 s, 3H, 3-CNCH_3_ (*E+Z*)), 3.00, 3.04 (2 d, ^2^*J* = 16.8 Hz, 1H, H-12_a_ (*E+Z*)), 3.62 (dd, ^2^*J* = 16.8 Hz, ^3^*J* = 6.1 Hz, 1H, H-12_b_ (*E+Z*)), 5.39 (d, ^3^*J* = 6.1 Hz, 1H, H-11), 6.71 (d, ^3^*J* = 7.6 Hz, 1H, H-7), 6.87 (t, ^3^*J* = 6.9 Hz, 1H, H-9), 7.03 (d, *J* = 7.6 Hz, 1H, H-10), 7.08–7.11 (m, 1H, H-8), 7.55–7.66 (m, 3H, H-3′,5′ Py, H-4), 8.70, 8.79 (2 br. s, 2H, H-2′,6′ Py (*E+Z*)), 9.16, 9.54 (2 br. s, 1H, N-H (*E+Z*)). ^13^C NMR (101 MHz, CDCl_3_) δ ppm 22.3, 25.5, 26.2, 39.4, 64.5 69.7, 96.6, 116.9, 120.6, 121.2, 121.6, 123.5, 125.8, 126.2, 128.8, 129.5, 130.8, 132.1, 133.6, 140.5, 149.8, 150.8, 150.9, 156.1. MS (Q-TOF) *m*/*z*: calcd for C_24_H_23_N_4_O_3_^+^ (*E+Z*) [M + H]^+^: 415.1725; found: 415.2477 (t_R_ = 5.449 min), 415.7866 (t_R_ = 5.715 min).

(*E*)-*N*′-(1-(2,5-dimethyl-11,12-dihydro-5*H*-5,11-epoxybenzo[7,8]oxocino[4,3-*b*]pyridin-3-yl)ethylidene)-2-hydroxybenzohydrazide **4b**. Yield: 765 mg (73%); White crystals, mp. 150–152 °C (2-PrOH). IR (ν_max_, cm^−1^) 3274 (-NH), 1643 (C=O), 1231 (-C-O-C-). ^1^H NMR (400 MHz, CDCl_3_) δ ppm 1.93 (s, 3H, 5-CH_3_), 2.29 (s, 3H, 2-CH_3_), 2.51 (s, 3H, 3-CNCH_3_), 3.03 (d, ^2^*J* = 16.8 Hz, 1H, H-12_a_), 3.62 (dd, ^2^*J* = 16.8, ^3^*J* = 6.1 Hz, 1H, H-12_b_), 5.38 (d, ^3^*J* = 6.1 Hz, 1H, H-11), 6.72 (d, ^3^*J* = 9.2 Hz, 1H, H-7), 6.84–6.91 (m, 2H, H-9, H-3′ Ar), 7.00–7.02 (m, 2H, H-10, H-5′ Ar), 7.08 (t, ^3^*J* = 7.6 Hz, 1H, H-8), 7.15 (t, ^3^*J* = 7.6 Hz, 1H, H-4’ Ar), 7.36 (m, 1H, H-6′ Ar), 7.70 (s, 1H, H-4), 7.79 (br. s, 1H, N-H), 10.19 (br. s, 1H, OH). ^13^C NMR (101 MHz, CDCl_3_) δ ppm 22.8, 23.3, 24.6, 26.2, 39.0, 69.6, 96.0, 96.6, 117.0, 118.4, 119.6, 120.3, 121.2, 121.6, 123.1, 125.7, 128.9, 129.2, 129.6, 133.7, 134.0, 134.7, 150.9, 152.2, 156.1. MS (Q-TOF) *m*/*z*: calcd for C_25_H_24_N_3_O_4_^+^ [M + H]^+^: 430.1722; found: 430.2949.

(*Z*)-4-(2,5-dimethyl-11,12-dihydro-5*H*-5,11-epoxybenzo[7,8]oxocino[4,3-*b*]pyridin-3-yl)-2-hydroxy-4-oxobut-2-enoic acid **5**. To a freshly prepared *n*-BuONa, prepared from sodium metal (700 mg, 30.0 mmol) dissolved in anhydrous *n*-BuOH (2.7 mL), we added diethyl oxalate (4.4 mL, 30.0 mmol) in anhydrous Et_2_O (50 mL). To this mixture, we added, with stirring, a solution of oxocino[4,3-*b*]pyridine **3** (5.95 g, 20.0 mmol) in anhydrous benzene (10 mL). The reaction mixture was refluxed for 8 h. The precipitate of diketo ester salt that formed after the completion of reaction was filtered, washed with anhydrous Et_2_O, and dissolved in 0.03 N NaOH (250 mL). The mixture was vigorously stirred for 3 h at room temperature, and then was acidified with concentrated HCl (to pH 4–5). The resulting precipitate was filtered, washed with water, and air-dried.

(*Z*)-4-(2,5-dimethyl-11,12-dihydro-5*H*-5,11-epoxybenzo[7,8]oxocino[4,3-*b*]pyridin-3-yl)-2-hydroxy-4-oxobut-2-enoic acid **5**. Yield: 5.11 g (70%); beige crystals, mp. 178–180 °C (2-PrOH/H_2_O 3:1). IR (ν_max_, cm^−1^) 1719 (C=O), 1230 (-C-O-C-). ^1^H NMR (400 MHz, CDCl_3_) δ ppm 1.97 (s, 3H, 5-CH_3_), 2.71 (s, 3H, 2-CH_3_), 3.21 (d, ^2^*J* = 16.8 Hz, 1H, H-12_a_), 3.71 (dd, ^2^*J* = 16.8 Hz, ^3^*J* = 6.1 Hz, 1H, H-12_b_), 5.42 (d, ^3^*J* = 6.1 Hz, 1H, H-11), 6.75 (d, ^3^*J* = 9.2 Hz, 1H, H-7), 6.84 (s, 1H, =CH), 6.90 (t, ^3^*J* = 7.6 Hz, 1H, H-9), 7.05 (d, ^3^*J* = 7.6 Hz, 1H, H-10), 7.11 (t, ^3^*J* = 7.6 Hz, 1H, H-8), 8.07 (s, 1H, H-4). ^13^C NMR (101 MHz, CDCl_3_) δ ppm 22.5, 26.0, 38.0, 68.8, 95.9, 101.0, 116.9, 121.5, 122.4, 125.7, 129.0, 130.8, 131.0, 135.6, 150.3, 154.6, 157.1, 163.9, 172.4, 191.1. MS (Q-TOF) *m*/*z*: calcd for C_20_H_18_NO_6_^+^ [M + H]^+^: 368.1089; found: 368.1644.

General procedure for the synthesis of 3,4-dihydroquinoxalin-2(1*H*)-one **6a** and 3,4-dihydro-2*H*-benzo[*b*][1,4]oxazin-2-ones **6b**–**d**. A mixture both of compound **5** (367 mg, 1.0 mmol) and 2-phenylenediamine or 2-aminophenol (1.0 mmol) in 2-PrOH (2 mL) was refluxed for 5 h and then left to cool. The formed yellow precipitate was filtered, washed with 2-PrOH, and air-dried.

(*Z*)-3-(2-(2,5-dimethyl-11,12-dihydro-5*H*-5,11-epoxybenzo[7,8]oxocino[4,3-*b*]pyridin-3-yl)-2-oxoethylidene)-3,4-dihydroquinoxalin-2(1*H*)-one **6a**. Yield: 246 mg (56%) yellow crystals, mp. 208–210 °C (2-PrOH/hexane 1:1). IR (ν_max_, cm^−1^) 1677 (C=O), 1219 (-C-O-C-). ^1^H NMR (400 MHz, CDCl_3_) δ ppm 1.95 (s, 3H, 5-CH_3_), 2.65 (s, 3H, 2-CH_3_), 3.09 (d, *J* = 16.8 Hz, 1H, H-12_a_), 3.67 (dd, ^2^*J* = 18.3 Hz, ^3^*J* = 6.1 Hz, 1H, H-12_b_), 5.42 (d, ^3^*J* = 6.1 Hz, 1H, H-11), 5.97 (s, 1H, =CH), 6.71 (d, ^3^*J* = 7.6 Hz, 1H, H-7), 6.89 (t, ^3^*J* = 7.6 Hz, 1H, H-9 oxocine), 7.03–7.12 (m, 3H, H-8,10, H-5′ Ar), 7.22–7.30 (m, 3H, H-6′,7′,8′ Ar), 7.82 (s, 1H, H-4), 8.36 (s, 1H, N_1_-H). 14.93 (s, 1H, -N_4_-H). ^13^C NMR (101 MHz, CDCl_3_) δ ppm 23.5, 26.2, 39.3, 69.6, 91.0, 96.5, 116.77, 116.8, 117.2, 121.1, 122.9, 123.1, 125.6, 125.7, 126.0, 128.7, 128.8, 129.2, 132.8, 134.9, 146.0, 150.7, 153.2, 155.3, 156.2, 191.2. MS (Q-TOF) *m*/*z*: calcd for C_26_H_22_N_3_O_4_^+^ [M + H]^+^: 440.1566; found: 440.2040.

(*Z*)-3-(2-(2,5-dimethyl-11,12-dihydro-5*H*-5,11-epoxybenzo[7,8]oxocino[4,3-*b*]pyridin-3-yl)-2-oxoethylidene)-3,4-dihydro-2*H*-benzo[*b*][1,4]oxazin-2-one **6b**. Yield: 290 mg (66%); yellow crystals, mp. 210–212 °C (2-PrOH/hexane 1:1). IR (ν_max_, cm^−1^) 1761 (C=O), 1286 (-C-O-C-). ^1^H NMR (400 MHz, CDCl_3_) δ ppm 1.97 (s, 3H, 5-CH_3_), 2.66 (s, 3H, 2-CH_3_), 3.07 (d, ^2^*J* = 17.4 Hz, 1H, H-12_a_), 3.66 (dd, ^2^*J* = 17.4 Hz, ^3^*J* = 5.5 Hz, 1H, H-12_b_), 5.41 (d, ^3^*J* = 5.5 Hz, 1H, H-11), 6.68 (s, 1H, =CH), 6.76 (d, ^3^*J* = 8.2 Hz, 1H, H-7), 6.88 (t, ^3^*J* = 7.3 Hz, 1H, H-9), 7.05 (d, ^3^*J* = 6.4 Hz, 1H, H-10), 7.11–7.17 (m, 3H, H-8, H-5′,6′ Ar), 7.20–7.24 (m, 2H, H-7′,8′ Ar), 7.91 (s, 1H, H-4). ^13^C NMR (101 MHz, CDCl_3_) δ ppm 23.9, 26.3, 39.5, 69.6, 96.6, 97.4, 116.3, 117.0, 117.3, 121.24, 123.1, 123.4, 124.6, 125.7, 126.1, 128.9, 129.5, 133.5, 133.9, 139.3, 141.5, 150.9, 154.3, 155.9, 157.30, 194.0. MS (Q-TOF) *m*/*z*: calcd for C_26_H_21_N_2_O_5_^+^ [M + H]^+^: 441.1406; found: 441.1656.

(*Z*)-3-(2-(2,5-dimethyl-11,12-dihydro-5*H*-5,11-epoxybenzo[7,8]oxocino[4,3-*b*]pyridin-3-yl)-2-oxoethylidene)-7-nitro-3,4-dihydro-2*H*-benzo[*b*][1,4]oxazin-2-one 6c. Yield: 204 mg (42%); yellow crystals, mp. 170–172 °C (2-PrOH/hexane 1:1). IR (ν_max_, cm^−1^) 1776 (C=O), 1577, 1332 (NO_2_), 1259 (-C-O-C-). ^1^H NMR (400 MHz, CDCl_3_) δ ppm 1.98 (s, 3H, 5-CH_3_), 2.66 (s, 3H, 2-CH_3_), 3.09 (d, ^2^*J* = 18.3 Hz, 1H, H-12_a_), 3.67 (dd, ^2^*J* = 16.8 Hz, ^3^*J* = 6.1 Hz, 1H, H-12_b_), 5.42 (d, ^3^*J* = 6.1 Hz, 1H, H-11), 6.77 (d, ^3^*J* = 7.6 Hz, 1H, H-7), 6.84–6.93 (m, 2H, =CH, H-9), 7.05 (d, ^3^*J* = 7.6 Hz, 1H, H-10), 7.12 (t, ^3^*J* = 7.6 Hz, 1H, H-8), 7.24 (d, ^3^*J* = 7.7 Hz, 1H, H-5′ Ar), 7.96 (s, 1H, H-4), 8.03–8.24 (m, 2H, H-6′, 8′ Ar), 12.90 (s, 1H, N-H). ^13^C NMR (101 MHz, CDCl_3_) δ ppm 23.8, 26.3, 39.4, 69.5, 96.4, 100.3, 113.6, 116.0, 117.0, 121.4, 121.9, 122.9, 125.8, 129.1, 129.1, 129.9, 133.0, 134.0, 137.5, 140.5, 143.2, 150.7, 154.7, 155.1, 157.7, 194.6. MS (Q-TOF) *m*/*z*: calcd for C_26_H_20_N_3_O_7_^+^ [M + H]^+^: 486.1493; found: 486.1534.

(*Z*)-3-(2-(2,5-dimethyl-11,12-dihydro-5*H*-5,11-epoxybenzo[7,8]oxocino[4,3-*b*]pyridin-3-yl)-2-oxoethylidene)-3,4-dihydro-2*H*-pyrido [3,2-*b*][1,4]oxazin-2-one **6d**. Yield: 231 mg (64%); yellow crystals, mp. 195–198 °C (CH_2_Cl_2_/hexane 1:2). IR (ν_max_, cm^−1^) 1773 (C=O), 1216 (-C-O-C-). ^1^H NMR (500 MHz, DMSO-*d_6_*) δ ppm 1.96 (s, 3H, 5-CH_3_), 2.55 (s, 3H, 2-CH_3_), 3.01 (d, ^2^*J* = 17.4 Hz, 1H, H-12_a_), 3.56 (dd, ^2^*J* = 17.5, ^3^*J* = 5.6 Hz, 1H, H-12_b_), 5.50 (d, ^3^*J* = 5.5 Hz, 1H, H-11), 6.58 (s, 1H, =CH), 6.75 (dd, ^3^*J* = 8.2, ^4^*J* = 1.1 Hz, 1H, H-7), 6.90 (td, ^3^*J* = 7.5, ^4^*J* = 1.1 Hz, 1H, H-9), 7.11 (td, ^3^*J* = 7.5, ^4^*J* = 1.6 Hz, 1H, H-8), 7.21–7.24 (m, 2H, H-10, H-7′ Ar), 7.73 (dd, ^3^*J* = 8.1, ^4^*J* = 1.4 Hz, 1H, H-8′ Ar), 8.11 (s, 1H, H-4), 8.19 (dd, ^3^*J* = 4.8, ^4^*J* = 1.4 Hz, 1H, H-6′ Ar), 12.59 (s, 1H, -NH). ^13^C NMR (126 MHz, DMSO-*d_6_*) δ ppm 23.3, 25.7, 38.9, 68.6, 96.4, 97.4, 116.1, 119.8, 121.0, 123.4, 124.1, 126.1, 128.7, 129.0, 133.1, 133.4, 137.3, 137.5, 140.4, 144.3, 150.4, 154.0, 155.0, 156.3, 193.6. MS (Q-TOF) *m*/*z*: calcd for C_25_H_20_N_3_O_5_^+^ [M + H]^+^: 442.1358; found: 442.1777.

### 4.2. Biological Tests

#### 4.2.1. Cell Culture and Virus Strain

Vero E6 (ATCC CRL-1586) cells were obtained from the collection of the National Center for Biotechnology (Nur-Sultan, Kazakhstan). Vero E6 cells were grown in DMEM with high glucose (Lonza BE12-604 F/U1) containing 10% FBS (Gibco Cat# 16000-044), 2 mM L-glutamine, 1% MEM vitamin solution (ThermoScientific Cat# 11120052), 1% non-essential amino acids (ThermoScientific Cat# 11140050), penicillin (100 U/mL), streptomycin (100 µg/mL).

The SARS-CoV-2 virus strain used in this work was obtained from a clinical sample by the authors themselves and registered under the name hCoV-19/Kazakhstan/20679/2020. The nucleotide sequence of the whole genome of this strain was determined, and the results are published in [41]. The nucleotide sequence of this virus genome was deposited in the GISAID database (https://www.gisaid.org/, accessed on 17 January 2022) with the number EPI_ISL_454501. The strain of coronavirus used belongs to the B1 phylogenetic line; isolates of this line were frequently encountered in the first half of 2020.

#### 4.2.2. Virus Stocks

The method described in [42] was used. Vero E6 cells were seeded in P100 dishes, 2 × 10^6^ cells per dish. Cultures were grown to 90% confluence (~8 × 10^6^ cells). To infect monolayers and produce virus stocks, a medium with the addition of 2% heat-inactivated FBS was used. Standard medium was changed to medium with 2% FBS before adding virus inoculum. The multiplicity of infection (MOI, the ratio of the number of viral particles to the number of cells in culture at the time of infecting) was 0.01.

Dishes with cultures were incubated in a CO_2_ incubator for 72 h. The cytopathic effect was monitored microscopically. Medium containing the virus was collected 72 h after infection. The medium was clarified by centrifugation, separated into 0.5 mL aliquots, and stored at −80 °C.

#### 4.2.3. Virus Titers

The limiting dilution (Reed–Muench) method was employed [43]. Vero E6 cells were seeded in 96-well plates (37,500 cells per well). Serial dilutions of the SARS-CoV-2 virus preparation were made in phosphate-buffered saline (PBS) supplemented with 1% heat-inactivated horse serum. Eight tenfold dilutions were made, from 1:10 to 1:10^8^. The dilutions were distributed in long rows of the plate (in wells 1–11). Row 12 was filled with medium without virus and served as an uninfected control. The plate was incubated for 1 h in a CO_2_ incubator. Virus inocula were removed and the wells were filled with 150 µL of medium with 2% FBS. The plates were incubated for 3–4 days until the CPE was apparent. The wells with monolayers exhibiting the CPE per row of the plate were counted. The results were processed according to the Reed–Muench scheme [44].

#### 4.2.4. Control Drug

Tilorone (2,7-bis[2-(diethylamino)ethoxy]fluoren-9-one hydrochloride) is a registered drug in the authors’ countries, used as a broad-spectrum antiviral. Tilorone is active against the SARS-CoV-2 virus; references are given in the Results section. Tilorone (Sigma Cat# 220957) was dissolved in growth medium to 200 μg/mL and sterilized by filtering through a 0.22 μm filter.

#### 4.2.5. Cytotoxicity Test

The cytotoxic effect of compounds was measured by determining the half-maximal inhibitory concentration, IC_50_ [45]. Vero E6 cells were seeded in 96-well plates, 20,000 cells per well. The plates were incubated overnight in a CO_2_ incubator. Compounds to be tested were dissolved in DMSO at 20 mg/mL. The solutions in DMSO were diluted 1:100 with growth medium so that the resulting concentration of the compound was 200 μg/mL and DMSO concentration was 1%. The resulting samples were used to fill row H in a plate with grown Vero E6 cells. Then, 150 μL of this sample was supplemented into wells 1–10 (row H). Next, 100 µL aliquots of the growth medium were distributed into rows A-G (wells 1–10). Then, 50 µL portions of the sample were transferred from a row to which the compound was recently added into the previous row (e.g., from H to G, from G to F, etc.). Two rows on each plate (rows 11–12) did not contain compounds and served as controls of normal cells.

To produce quantitative results, live cell staining with nitrosine tetrazolium (Thiazolyl Blue Tetrazolium Bromide, MTT, Sigma Cat# M2128) was used. This test exploits the ability of MTT to stain only living cells, in which cellular oxidoreductases convert MTT to a purple-colored formazan. Aliquots (50 µL) of MTT solution (3 mg/mL) in medium without serum were added to the wells. The plate was incubated for 3 h. The existing medium was removed and formazan was dissolved in 100 μL of DMSO acidified with 1% glacial acetic acid. Optical densities were read using a plate photometer at 595 nm. The experiment was performed in duplicate for each compound. The IC_50_ values were obtained by interpolating optical densities using four-parameter non-linear regression [46].

#### 4.2.6. Antiviral Activity

The antiviral activity was measured by determining the compound concentration, which, in a limiting-dilution-format assay, reduces counts of infected wells per row by 50%. The method is published in [47]. First, 96-well plates with growing Vero E6 cells were filled with compounds diluted in growth medium in much the same way as described above in the section “Cytotoxicity Test”. Immediately after this, the SARS-CoV-2 virus was added to rows A-H (wells 1–11) in a volume of 2000 viral particles per well (MOI = 0.1). Row 11 in each plate did not contain compounds and was infected with the virus; this row was the virus growth control. Row 12 in each plate did not contain compounds and was not infected; this row was the control of normal cells.

The appearance of the CPE was monitored during 3–4-days of observation. The wells with the CPE were counted and the counts were used to determine the concentration that yielded a 50% effect (EC_50_) utilizing the Reed–Muench computation scheme. The experiment was performed in duplicate for each compound.

### 4.3. Data Evaluation

Data processing was carried out in GraphPad Prism [48]. Descriptive statistics were computed and mean values were used for graphs. Four-parameter non-linear regression was used to obtain IC_50_ values.

## Figures and Tables

**Table 1:** Cytotoxicity and antiviral activity against SARS-CoV-2 in Vero E6 cells.

Compound	Structure	IC_50_, µg/mL *	EC_50_, µg/mL *
**3**	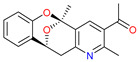	131.1	>200
**4a**	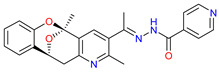	>200	>200
**4b**	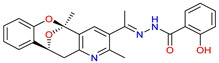	>200	>200
**5**	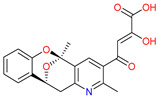	>200	>200
**6a**	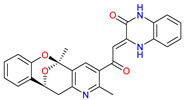	6.0	2.2
**6b**	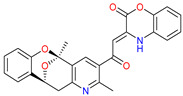	>200	>200
**6c**	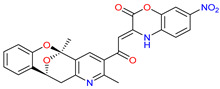	>200	>200
Tilorone **	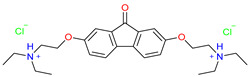	7.8	4.3

Notes: * The upper limit of concentrations tested is 200 µg/mL. If the value exceeds the upper limit of the tested concentrations, “>200” is indicated. ** Tilorone (2,7-bis(2-diethylamino)ethoxy)fluoren-9-one dihydrochloride) is a registered pharmaceutical drug in the authors’ countries and, despite not being recommended for the treatment of patients with COVID-19, has proven activity against SARS-CoV-2 in Vero E6 cells. In this study, tilorone was used as a control with proven activity against SARS-CoV-2.

**Table 2:** Docking score values of compound **6a** and reference drug (tilorone) for target protein 7AAP, kcal/mol.

	Receptor	7AAP
Ligand		
**6a**		−9.7
Tilorone		−6.7

## Data Availability

Not applicable.

## Supplementary Materials

The following supporting information can be downloaded at: https://www.mdpi.com/article/10.3390/molecules27123701/s1. S1–S5, Experimental Procedures; S6–S12, ^1^H and ^13^C NMR spectra; S13–S15, Mass spectra; S16–S23, Biological test procedure.

## Sample Availability

Samples of the compounds are available from the authors.
