# Supplementary material for: Synthesis and Antiviral Properties against SARS-CoV-2 of Epoxybenzooxocino[4,3-b]Pyridine Derivatives
Source: Molecules. 2022 Jun 9;27(12):3701. doi: 10.3390/molecules27123701 (PMC9230803; doi:10.3390/molecules27123701)
Supplement: Supplementary file 1 [file molecules-27-03701-s001.zip › molecules-1696400-supplementary.pdf]

Supporting information

Synthesis and antiviral properties against SARS-CoV-2 of epoxybenzooxocino[4,3-b]pyridine derivatives

Alena L. Stalinskaya[a], Nadezhda V. Martynenko[a], Zarina T. Shulgau[b]

Alexandr V. Shustov[b], Viktoriya V. Keyer[b], Ivan V. Kulakov[a,b*]

Table of contents

Experimental 1

General Information 1

Experimental Procedures 2

Spectroscopic and physical data 2

Copies of NMR Spectra of Products 6

Copies of MS Spectra of Products 13

Biological tests 16

Experimental

General Information

FTIR spectra were obtained with an Agilent Cary 630 spectrophotometer in a thin sample layer on a crystal attachment. ^{1}H and ^{13}C NMR spectra were recorded on a Bruker DRX400 (400 and 100 MHz, respectively) and Bruker AVANCE 500 (500 and 125 MHz, respectively) instruments using DMSO-d_6 (compound **6d**) or $CDCl_3$ (remaining compounds) the internal standard was TMS or residual solvent signals (7.25 and 77.0 ppm in the case of $CDCl_3$ for ^{1}H and ^{13}C nuclei, respectively; 2.49 and 39.9 ppm ^{1}H and for ^{13}C nuclei in DMSO-d_6).

Sample were analyzed by HPLC-MS on an Agilent 1260 Infinity II chromatograph coupled to an Agilent 6545 LC/Q-TOF high-resolution mass spectrometer with a Dual AJS ESI ionization source operating in positive ion mode using the following parameters: capillary voltage: 4000 V; spray pressure: 20 (psi); drying gas: 10 l/min; gas temperature: 325 ºC; sheathed gas flow: 12 l/min; shielding gas temperature: 400 °C; nozzle voltage: 0 V, fragmentation voltage: 180 V; skimmer voltage: 45 V; octopole RF: 750 V. Mass spectra with LC/MS accuracy were recorded in the range 100-1000 m/z, scan rate 1.5 spectrum/s.

Chromatographic separation was carried out on columns: ZORBAX RRHD Eclipse Plus C18 (2.1 x 50 mm, particle size 1.8 μm). The column temperature during the analysis was maintained at 35 ° C. The mobile phase was formed by eluents A and B. In the positive ionization mode, 0.1% formic acid solution in deionized water was used as eluent A, and 0.1% formic acid solution in acetonitrile was used as eluent B. Chromatographic separation was performed with elution according to the following scheme: 0-10 min 95% A, 10-13 min 100% B, 13-15 min 95% A. The flow of the mobile phase was maintained at 400 μL/min throughout the analysis. In all experiments, the sample injection volume was 1 μL. The sample was prepared by dissolving the entire sample (in 1000 μL) in methanol (for HPLC). Sample dilution was carried out immediately before analysis.

The recorded data were processed using Agilent MassHunter 10.0 software.

Melting points were determined using a Stuart SMP10 hot bench. Monitoring of the reaction course and the purity of the products was carried out by TLC on Sorbfil plates and visualized using iodine vapor or UV light.

Experimental Procedures

1-(2,5-dimethyl-11,12-dihydro-5H-5,11-epoxybenzo[7,8]oxocino[4,3-b]pyridin-3-yl)ethan-1-one 3 was synthesized according to published procedures.[1]

General procedure for the synthesis of N-(ethan-1-yl-1-ylidene)benzohydrazides 4a,b. A solution of 1-(2,5-dimethyl-11,12-dihydro-5*H*-5,11-epoxybenzo[7,8]oxocino[4,3-*b*]pyridin-3-yl)ethan-1-one **3** (478 mg, 2.5 mmol) in 2-PrOH was treated with the appropriate hydrazide (5.0 mmol) and 1 drop of formic acid. The reaction mixture was heated at reflux for 5 h. The formed white precipitate was filtered off, washed with 2-PrOH, and air-dried.

(Z)-4-(2,5-dimethyl-11,12-dihydro-5H-5,11-epoxybenzo[7,8]oxocino[4,3-b]pyridin-3-yl)-2-hydroxy-4-oxobut-2-enoic acid 5. To a freshly prepared *n*-BuONa, prepared from sodium metal (700 mg, 30.0 mmol) dissolved in anhydrous *n*-BuOH (2.7 mL), was added diethyl oxalate (4.4 g, 30.0 mmol) in anhydrous Et_2O (50 mL). To this mixture was added with stirring a solution of 1-(2,5-dimethyl-11,12-dihydro-5*H*-5,11-epoxybenzo[7,8]oxocino[4,3-*b*]pyridin-3-yl)ethan-1-one **3** (5.95 g, 20.0 mmol) in anhydrous benzene (10 ml). The reaction mixture was reflux for 8 h. The precipitate of diketo ester salt that formed after the completion of reaction was filtered, washed with anhydrous Et_2O and dissolved in 0.03 N NaOH (250 mL). The mixture was vigorously stirred for 3 h at room temperature, then was acidified with concentrated HCl (to pH 4–5). The resulting precipitate was filtered, washed with water, and air-dried.

General procedure for the synthesis of 3,4-dihydroquinoxalin-2(1H)-one 6a and 3,4-dihydro-2H-benzo[b][1,4]oxazin-2-ones 6b-d. A mixture both of compound **5** (367 mg, 1.0 mmol) and 2-phenylenediamine or 2-aminophenol (1.0 mmol) in 2-PrOH (2 mL) was refluxed for 5 h and then left to cool. The formed yellow precipitate was filtered, washed with 2-PrOH, and air-dried.

Spectroscopic and physical data

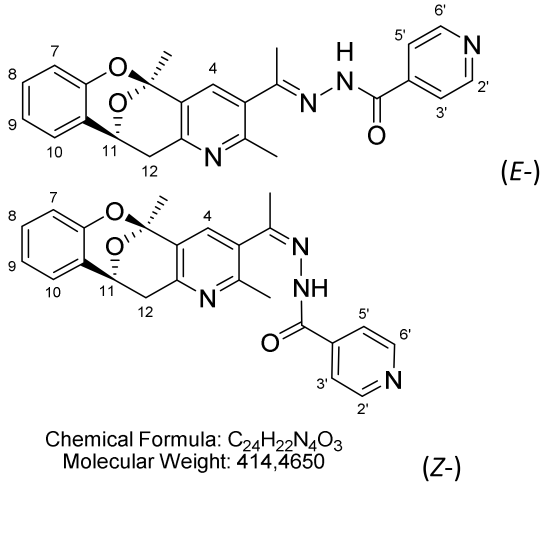	**(*E,Z*)-*N′*-(1-(2,5-dimethyl-11,12-dihydro-5*H*-5,11-epoxybenzo[7,8]oxocino[4,3-*b*]pyridin-3-yl)ethylidene)isonicotinohydrazide 4a.** Yield: 631 mg (62%); White crystals, mp. 244-246°C (2-PrOH). IR (ν_{max}, cm^{-1}) 3429 (-NH), 1675 (C=O), 1234 (-C-O-C-). ^{1}H NMR (400 MHz, $CDCl_3$) δ ppm 1.90, 1.93 (2 s, 3H, 5-CH_3 (*E*+*Z*)), 2.28, 2.31 (2 s, 3H, 2-CH_3 (*E*+*Z*)), 2.38, 2.53 (2 s, 3H, 3-$CNCH_3$ (*E*+*Z*)), 3.00, 3.04 (2 d, ^2J = 16.8 Hz, 1H, H-12$_a$ (*E*+*Z*)), 3.62 (dd, ^2J = 16.8 Hz, ^3J = 6.1 Hz, 1H, H-12$_b$ (*E*+*Z*)), 5.39 (d, ^3J = 6.1 Hz, 1H, H-11), 6.71 (d, ^3J = 7.6 Hz, 1H, H-7), 6.87 (t, ^3J = 6.9 Hz, 1H, H-9), 7.03 (d, *J* = 7.6 Hz, 1H, H-10), 7.08-7.11 (m, 1H, H-8), 7.55 – 7.66 (m, 3H, H-3′,5′ Py, H-4), 8.70, 8.79 (2 br. s, 2H, H-2′,6′ Py (*E*+*Z*)), 9.16, 9.54 (2 br. s, 1H, N-H (*E*+*Z*)). ^{13}C NMR (101 MHz, $CDCl_3$) δ ppm 22.3, 25.5, 26.2,

[1] Kulakov, I. V., Stalinskaya, A. L., Chikunov, S. Y., Gatilov, Y. V. (2021). Synthesis of new representatives of 11,12-dihydro-5*H*-5,11-epoxybenzo[7,8]oxocino[4,3-*b*]pyridines–structural analogues of integrastatins A, B. *New Journal of Chemistry*, 45(7), 3559-3569

	39.4, 64.5 69.7, 96.6, 116.9, 120.6, 121.2, 121.6, 123.5, 125.8, 126.2, 128.8, 129.5, 130.8, 132.1, 133.6, 140.5, 149.8, 150.8, 150.9, 156.1. MS (Q-TOF) m/z: calcd for $C_{24}H_{23}N_4O_3^+$ (E+Z) [M + H]$^+$: 415.1725; found: 415.2477 (t_R = 5.449 min), 415.7866 (t_R = 5.715 min).
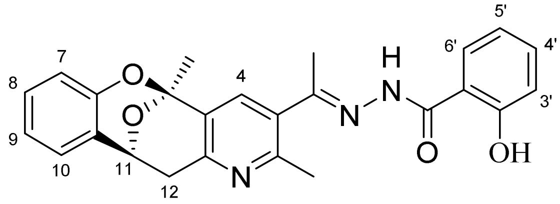<br>Chemical Formula: $C_{25}H_{23}N_3O_4$<br>Molecular Weight: 429,4760	**(*E*)-*N′*-(1-(2,5-dimethyl-11,12-dihydro-5*H*-5,11-epoxybenzo[7,8]oxocino[4,3-*b*]pyridin-3-yl)ethylidene)-2-hydroxybenzohydrazide 4b**. Yield: 765 mg (73%); White crystals, mp 150-152°C (2-PrOH). IR (ν_{max}, cm^{-1}) 3274 (-NH), 1643 (C=O), 1231 (-C-O-C-). ^{1}H NMR (400 MHz, $CDCl_3$) δ ppm 1.93 (s, 3H, 5-CH_3), 2.29 (s, 3H, 2-CH_3), 2.51 (s, 3H, 3-$CNCH_3$), 3.03 (d, ^2J = 16.8 Hz, 1H, H-12$_a$), 3.62 (dd, ^2J = 16.8, ^3J = 6.1 Hz, 1H, H-12$_b$), 5.38 (d, ^3J = 6.1 Hz, 1H, H-11), 6.72 (d, ^3J = 9.2 Hz, 1H, H-7), 6.84 – 6.91 (m, 2H, H-9, H-3′ Ar), 7.00-7.02 (m, 2H, H-10, H-5′ Ar), 7.08 (t, ^3J = 7.6 Hz, 1H, H-8), 7.15 (t, ^3J = 7.6 Hz, 1H, H-4' Ar), 7.36 (m, 1H, H-6′ Ar), 7.70 (s, 1H, H-4), 7.79 (br. s, 1H, N-H), 10.19 (br. s, 1H, OH). ^{13}C NMR (101 MHz, $CDCl_3$) δ ppm 22.8, 23.3, 24.6, 26.2, 39.0, 69.6, 96.0, 96.6, 117.0, 118.4, 119.6, 120.3, 121.2, 121.6, 123.1, 125.7, 128.9, 129.2, 129.6, 133.7, 134.0, 134.7, 150.9, 152.2, 156.1. MS (Q-TOF) m/z: calcd for $C_{25}H_{24}N_3O_4^+$ [M +H]$^+$: 430.1722; found: 430.2949
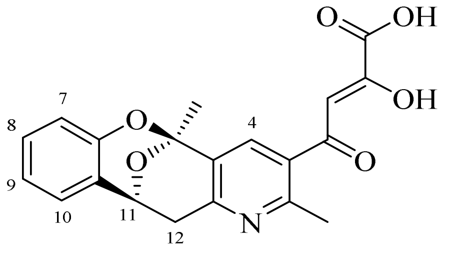<br>Chemical Formula: $C_{20}H_{17}NO_6$<br>Molecular Weight: 367,3570	**(*Z*)-4-(2,5-dimethyl-11,12-dihydro-5*H*-5,11-epoxybenzo[7,8]oxocino[4,3-*b*]pyridin-3-yl)-2-hydroxy-4-oxobut-2-enoic acid 5.** Yield: 5.11 g (70%); beige crystals, mp 178-180°C (2-PrOH/H_2O 3:1). IR (ν_{max}, cm^{-1}) 1719 (C=O), 1230 (-C-O-C-). ^{1}H NMR (400 MHz, $CDCl_3$) δ ppm 1.97 (s, 3H, 5-CH_3), 2.71 (s, 3H, 2-CH_3), 3.21 (d, ^2J = 16.8 Hz, 1H, H-12$_a$), 3.71 (dd, ^2J = 16.8 Hz, ^3J = 6.1 Hz, 1H, H-12$_b$), 5.42 (d, ^3J = 6.1 Hz, 1H, H-11), 6.75 (d, ^3J = 9.2 Hz, 1H, H-7), 6.84 (s, 1H, =CH), 6.90 (t, ^3J = 7.6 Hz, 1H, H-9), 7.05 (d, ^3J = 7.6 Hz, 1H, H-10), 7.11 (t, ^3J = 7.6 Hz, 1H, H-8), 8.07 (s, 1H, H-4). ^{13}C NMR (101 MHz, $CDCl_3$) δ ppm 22.5, 26.0, 38.0, 68.8, 95.9, 101.0, 116.9, 121.5, 122.4, 125.7, 129.0, 130.8, 131.0, 135.6, 150.3, 154.6, 157.1, 163.9, 172.4, 191.1. MS (Q-TOF) m/z: calcd for $C_{20}H_{18}NO_6^+$ [M + H]$^+$: 368.1089; found: 368.1644.
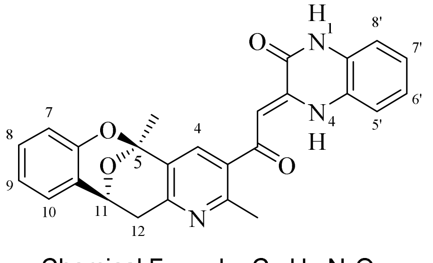<br>Chemical Formula: $C_{26}H_{21}N_3O_4$<br>Exact Mass: 439,1532	**(*Z*)-3-(2-(2,5-dimethyl-11,12-dihydro-5*H*-5,11-epoxybenzo[7,8]oxocino[4,3-*b*]pyridin-3-yl)-2-oxoethylidene)-3,4-dihydroquinoxalin-2(1*H*)-one 6a.** Yield: 246 mg (56%) yellow crystals, mp 208-210°C (2-PrOH/hexane 1:1). IR (ν_{max}, cm^{-1}) 1677 (C=O), 1219 (-C-O-C-). ^{1}H NMR (400 MHz, $CDCl_3$) δ ppm 1.95 (s, 3H, 5-CH_3), 2.65 (s, 3H, 2-CH_3), 3.09 (d, J = 16.8 Hz, 1H, H-12$_a$), 3.67 (dd, ^2J = 18.3 Hz, ^3J = 6.1 Hz, 1H, H-12$_b$), 5.42 (d, ^3J = 6.1 Hz, 1H, H-11), 5.97 (s, 1H, =CH), 6.71 (d, ^3J = 7.6 Hz, 1H, H-7), 6.89 (t, ^3J = 7.6

	Hz, 1H, H-9 oxocine), 7.03 – 7.12 (m, 3H, H-8,10, H-5′ Ar), 7.22 – 7.30 (m, 3H, H-6′,7′,8′ Ar), 7.82 (s, 1H, H-4), 8.36 (s, 1H, N_1-H). 14.93 (s, 1H, -N_4-H). ^{13}C NMR (101 MHz, $CDCl_3$) δ ppm 23.5, 26.2, 39.3, 69.6, 91.0, 96.5, 116.77, 116.8, 117.2, 121.1, 122.9, 123.1, 125.6, 125.7, 126.0, 128.7, 128.8, 129.2, 132.8, 134.9, 146.0, 150.7, 153.2, 155.3, 156.2, 191.2. MS (Q-TOF) m/z: calcd for $C_{26}H_{22}N_3O_4^+$ $[M + H]^+$: 440.1566; found: 440.2040.
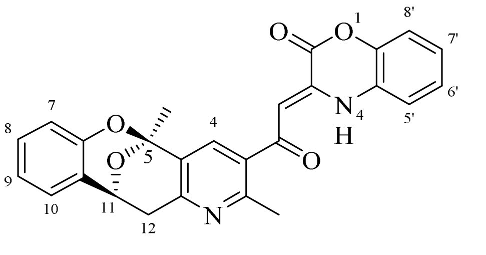Chemical Formula: $C_{26}H_{20}N_2O_5$<br>Exact Mass: 440,1372	**(*Z*)-3-(2-(2,5-dimethyl-11,12-dihydro-5*H*-5,11-epoxybenzo[7,8]oxocino[4,3-*b*]pyridin-3-yl)-2-oxoethylidene)-3,4-dihydro-2*H*-benzo[*b*][1,4]oxazin-2-one 6b.** Yield: 290 mg (66%); yellow crystals, mp 210-212°C (2-PrOH/hexane 1:1). IR (ν_{max}, cm^{-1}) 1761 (C=O), 1286 (-C-O-C-). ^{1}H NMR (400 MHz, $CDCl_3$) δ ppm 1.97 (s, 3H, 5-CH_3), 2.66 (s, 3H, 2-CH_3), 3.07 (d, ^2J = 17.4 Hz, 1H, H-12_a), 3.66 (dd, ^2J = 17.4 Hz, ^3J = 5.5 Hz, 1H, H-12_b), 5.41 (d, ^3J = 5.5 Hz, 1H, H-11), 6.68 (s, 1H, =CH), 6.76 (d, ^3J = 8.2 Hz, 1H, H-7), 6.88 (t, ^3J = 7.3 Hz, 1H, H-9), 7.05 (d, ^3J = 6.4 Hz, 1H, H-10), 7.11 – 7.17 (m, 3H, H-8, H-5′,6′ Ar), 7.20 – 7.24 (m, 2H, H-7′,8′ Ar), 7.91 (s, 1H, H-4). ^{13}C NMR (101 MHz, $CDCl_3$) δ ppm 23.9, 26.3, 39.5, 69.6, 96.6, 97.4, 116.3, 117.0, 117.3, 121.24, 123.1, 123.4, 124.6, 125.7, 126.1, 128.9, 129.5, 133.5, 133.9, 139.3, 141.5, 150.9, 154.3, 155.9, 157.30, 194.0. MS (Q-TOF) m/z: calcd for $C_{26}H_{21}N_2O_5^+$ $[M + H]^+$: 441.1406; found: 441.1656.
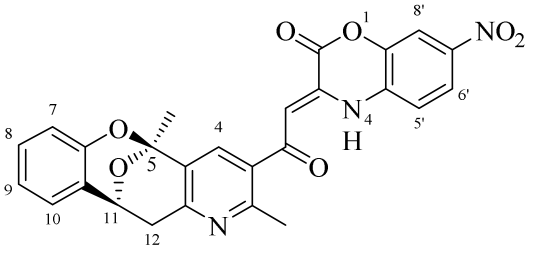Chemical Formula: $C_{26}H_{19}N_3O_7$<br>Exact Mass: 485,1223	**(Z)-3-(2-(2,5-dimethyl-11,12-dihydro-5H-5,11-epoxybenzo[7,8]oxocino[4,3-b]pyridin-3-yl)-2-oxoethylidene)-7-nitro-3,4-dihydro-2*H*-benzo[*b*][1,4]oxazin-2-one 6c.** Yield: 204 mg (42%); yellow crystals, mp 170-172°C (2-PrOH/hexane 1:1). IR (ν_{max}, cm^{-1}) 1776 (C=O), 1577, 1332 (NO_2), 1259 (-C-O-C-). ^{1}H NMR (400 MHz, $CDCl_3$) δ ppm 1.98 (s, 3H, 5-CH_3), 2.66 (s, 3H, 2-CH_3), 3.09 (d, ^2J = 18.3 Hz, 1H, H-12_a), 3.67 (dd, ^2J = 16.8 Hz, ^3J = 6.1 Hz, 1H, H-12_b), 5.42 (d, ^3J = 6.1 Hz, 1H, H-11), 6.77 (d, ^3J = 7.6 Hz, 1H, H-7), 6.84 – 6.93 (m, 2H, =CH, H-9), 7.05 (d, ^3J = 7.6 Hz, 1H, H-10), 7.12 (t, ^3J = 7.6 Hz, 1H, H-8), 7.24 (d, ^3J = 7.7 Hz, 1H, H-5′ Ar), 7.96 (s, 1H, H-4), 8.03 – 8.24 (m, 2H, H-6′,8′ Ar), 12.90 (s, 1H, N-H). ^{13}C NMR (101 MHz, $CDCl_3$) δ ppm 23.8, 26.3, 39.4, 69.5, 96.4, 100.3, 113.6, 116.0, 117.0, 121.4, 121.9, 122.9, 125.8, 129.1, 129.1, 129.9, 133.0, 134.0, 137.5, 140.5, 143.2, 150.7, 154.7, 155.1, 157.7, 194.6. MS (Q-TOF) m/z: calcd for $C_{26}H_{20}N_3O_7^+$ $[M + H]^+$: 486.1493; found: 486.1534.

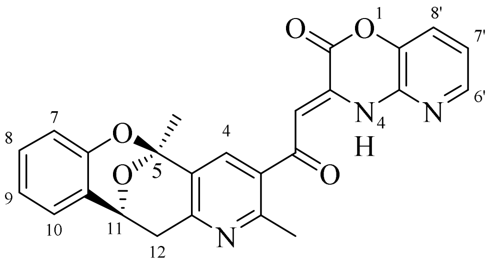 Chemical Formula: $C_{25}H_{19}N_3O_5$<br>Exact Mass: 441,1325	**(*Z*)-3-(2-(2,5-dimethyl-11,12-dihydro-5*H*-5,11-epoxybenzo[7,8]oxocino[4,3-*b*]pyridin-3-yl)-2-oxoethylidene)-3,4-dihydro-2*H*-pyrido[3,2-*b*][1,4]oxazin-2-one 6d.** Yield: 231 mg (64%); yellow crystals, mp 195-198°C (CH_2Cl_2/hexane 1:2). IR (ν_{max}, cm^{-1}) 1773 (C=O), 1216 (-C-O-C-). ^1H NMR (500 MHz, DMSO-d_6) δ ppm 1.96 (s, 3H, 5-CH_3), 2.55 (s, 3H, 2-CH_3), 3.01 (d, ^2J = 17.4 Hz, 1H, H-12_a), 3.56 (dd, ^2J = 17.5, ^3J = 5.6 Hz, 1H, H-12_b), 5.50 (d, ^3J = 5.5 Hz, 1H, H-11), 6.58 (s, 1H, =CH), 6.75 (dd, ^3J = 8.2, ^4J = 1.1 Hz, 1H, H-7), 6.90 (td, ^3J = 7.5, ^4J = 1.1 Hz, 1H, H-9), 7.11 (td, ^3J = 7.5, ^4J = 1.6 Hz, 1H, H-8), 7.21 – 7.24 (m, 2H, H-10, H-7′ Ar), 7.73 (dd, ^3J = 8.1, ^4J = 1.4 Hz, 1H, H-8′ Ar), 8.11 (s, 1H, H-4), 8.19 (dd, ^3J = 4.8, ^4J = 1.4 Hz, 1H, H-6′ Ar), 12.59 (s, 1H, -NH). ^{13}C NMR (126 MHz, DMSO-d_6) δ ppm 23.3, 25.7, 38.9, 68.6, 96.4, 97.4, 116.1, 119.8, 121.0, 123.4, 124.1, 126.1, 128.7, 129.0, 133.1, 133.4, 137.3, 137.5, 140.4, 144.3, 150.4, 154.0, 155.0, 156.3, 193.6. MS (Q-TOF) m/z: calcd for $C_{25}H_{20}N_3O_5^+$ $[M + H]^+$: 442.1358; found: 442.1777.

Copies of NMR Spectra of Products

	ppm	Hz	Hight		ppm	Hz	Hight
1	1.90	758.5	2.6	17	6.70	2678.1	1.7
2	1.93	772.3	2.1	18	6.72	2685.7	1.9
3	2.28	912.7	3.0	19	6.85	2739.1	0.7
4	2.31	921.8	2.2	20	6.87	2745.2	0.9
5	2.38	950.8	2.1	21	6.89	2752.9	0.4
6	2.53	1010.3	1.7	22	7.02	2807.8	1.5
7	2.99	1194.9	0.5	23	7.04	2815.4	1.6
8	3.01	1202.6	0.4	24	7.07	2826.1	0.8
9	3.03	1211.7	0.6	25	7.09	2833.7	0.8
10	3.05	1220.9	0.3	26	7.26	2902.4	1.9
11	3.58	1431.5	0.3	27	7.55	3019.9	0.7
12	3.60	1439.1	0.5	28	7.66	3062.6	2.1
13	3.64	1455.9	0.5	29	8.70	3476.1	1.0
14	3.66	1463.5	0.1	30	8.79	3512.8	0.8
15	5.38	2151.7	1.5	31	9.16	3662.3	0.3
16	5.40	2157.8	1.4	32	9.54	3814.9	0.3

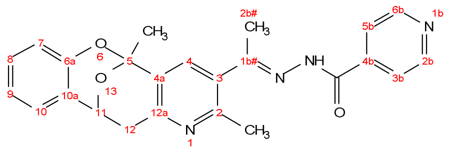

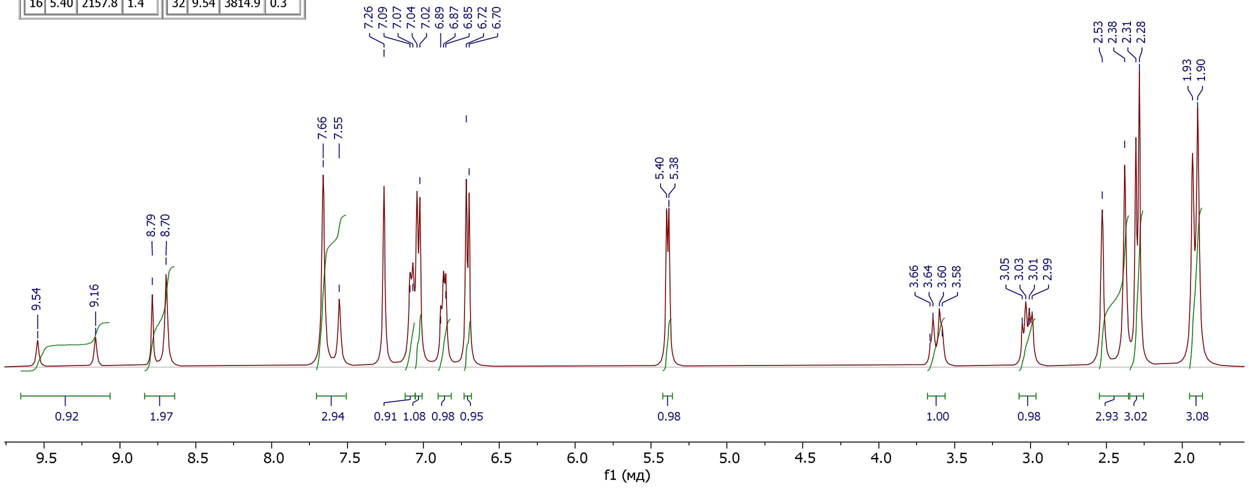

13C NMR (101 MHz, CHLOROFORM - *D*) δ 156.1, 150.9, 150.8, 140.5, 133.6, 132.1, 130.8, 129.5, 128.8, 126.1, 125.8, 123.2, 121.6, 121.2, 120.6, 116.9, 96.6, 69.7, 64.5, 39.4, 26.2, 25.5, 22.3.

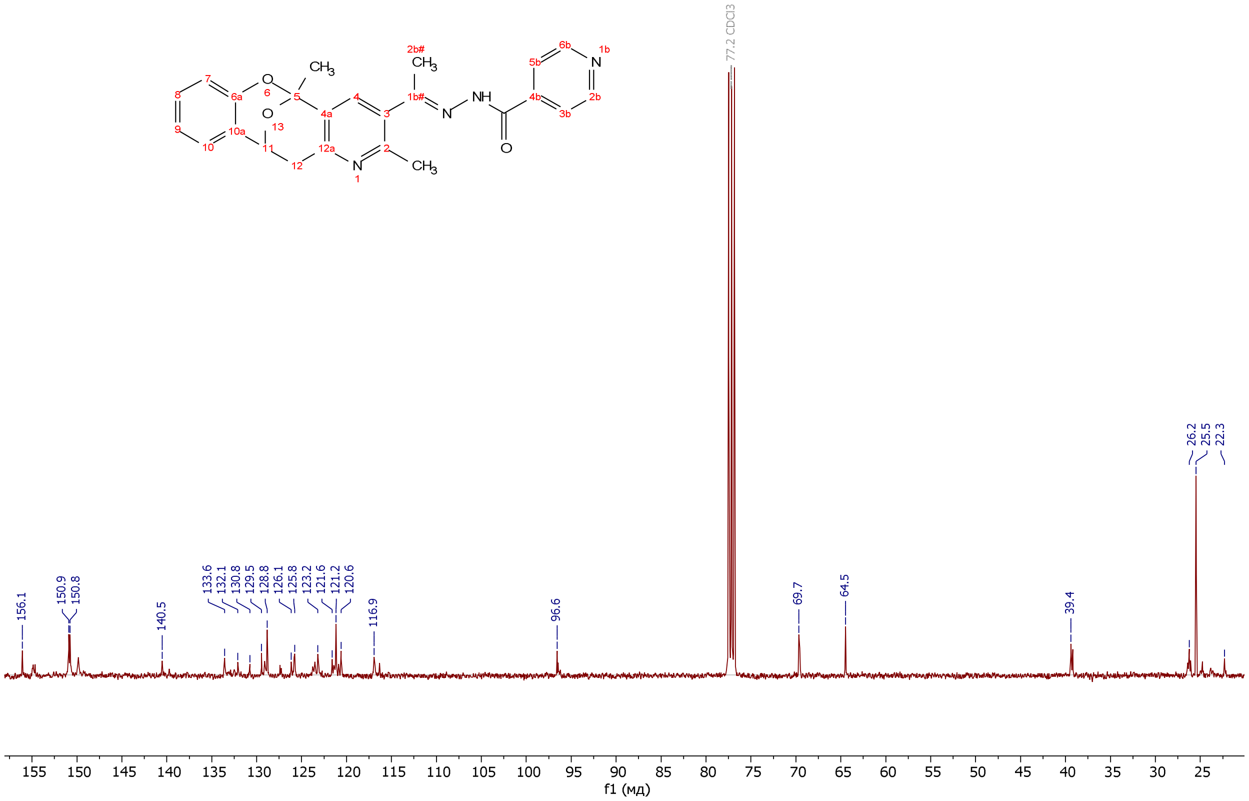

^{1}H (400 MHz, $CDCl_3$) and ^{13}C (100 MHz, $CDCl_3$) NMR Spectra of **4a**

	ppm	Hz	Hight		ppm	Hz	Hight
1	1.93	770.8	5.1	17	6.91	2763.6	0.6
2	2.29	914.2	4.2	18	7.00	2798.7	1.3
3	2.51	1002.7	4.7	19	7.02	2806.3	1.2
4	3.01	1202.6	0.7	20	7.07	2824.6	0.4
5	3.05	1219.4	0.8	21	7.08	2832.3	0.8
6	3.59	1434.6	0.4	22	7.10	2839.9	0.5
7	3.60	1440.7	0.4	23	7.13	2850.6	0.3
8	3.63	1451.3	0.4	24	7.15	2858.2	0.5
9	3.65	1457.4	0.5	25	7.17	2865.8	0.3
10	5.37	2147.1	0.9	26	7.26	2902.4	1.0
11	5.39	2153.2	0.8	27	7.34	2936.0	0.5
12	6.71	2681.2	0.9	28	7.36	2943.6	0.7
13	6.73	2690.4	1.0	29	7.38	2951.3	0.3
14	6.86	2740.7	0.9	30	7.70	3077.9	1.7
15	6.87	2748.3	1.2	31	7.79	3113.0	0.3
16	6.89	2756.0	1.2	32	10.19	4074.3	0.1

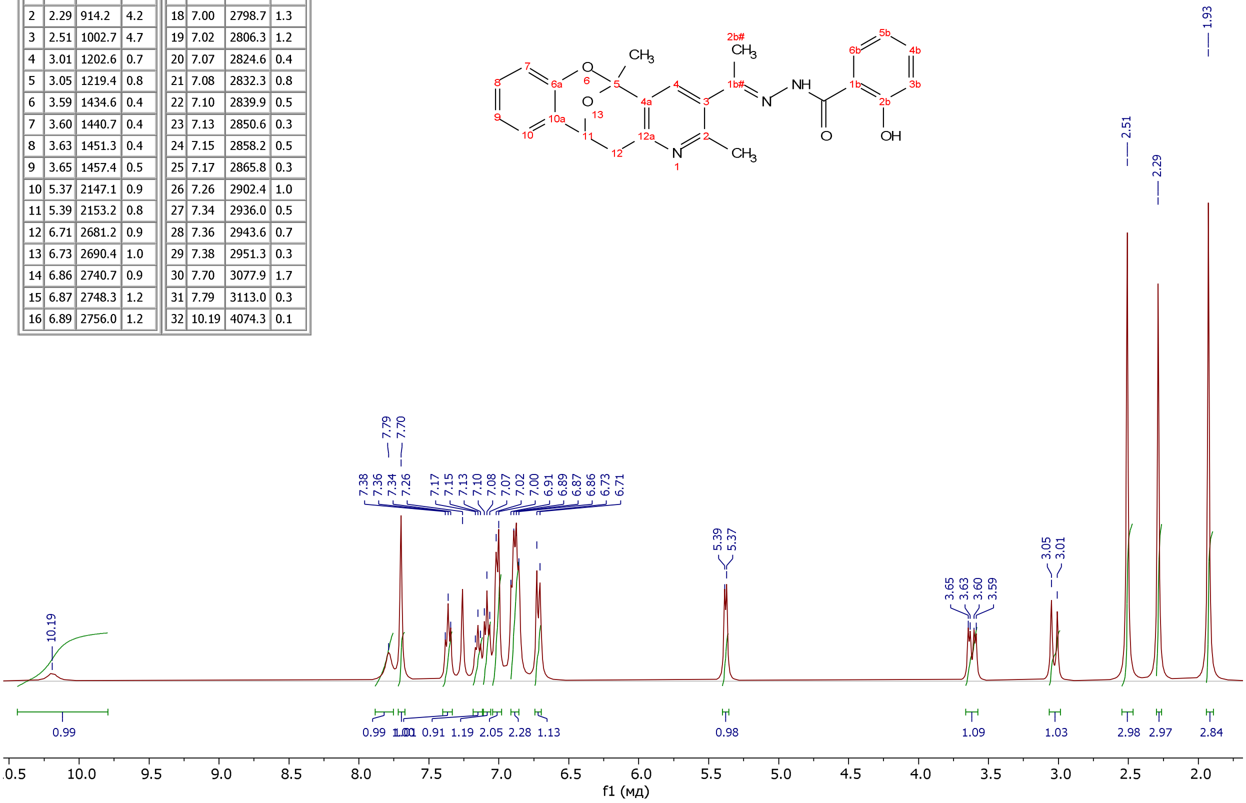

13C NMR (101 MHz, CHLOROFORM - *D*) δ 156.1, 152.2, 150.9, 134.7, 134.0, 133.7, 129.6, 129.2, 128.9, 125.7, 123.1, 122.5, 121.6, 121.2, 120.3, 119.5, 118.4, 117.0, 96.6, 96.0, 69.6, 39.0, 26.2, 24.6, 23.3, 22.8.

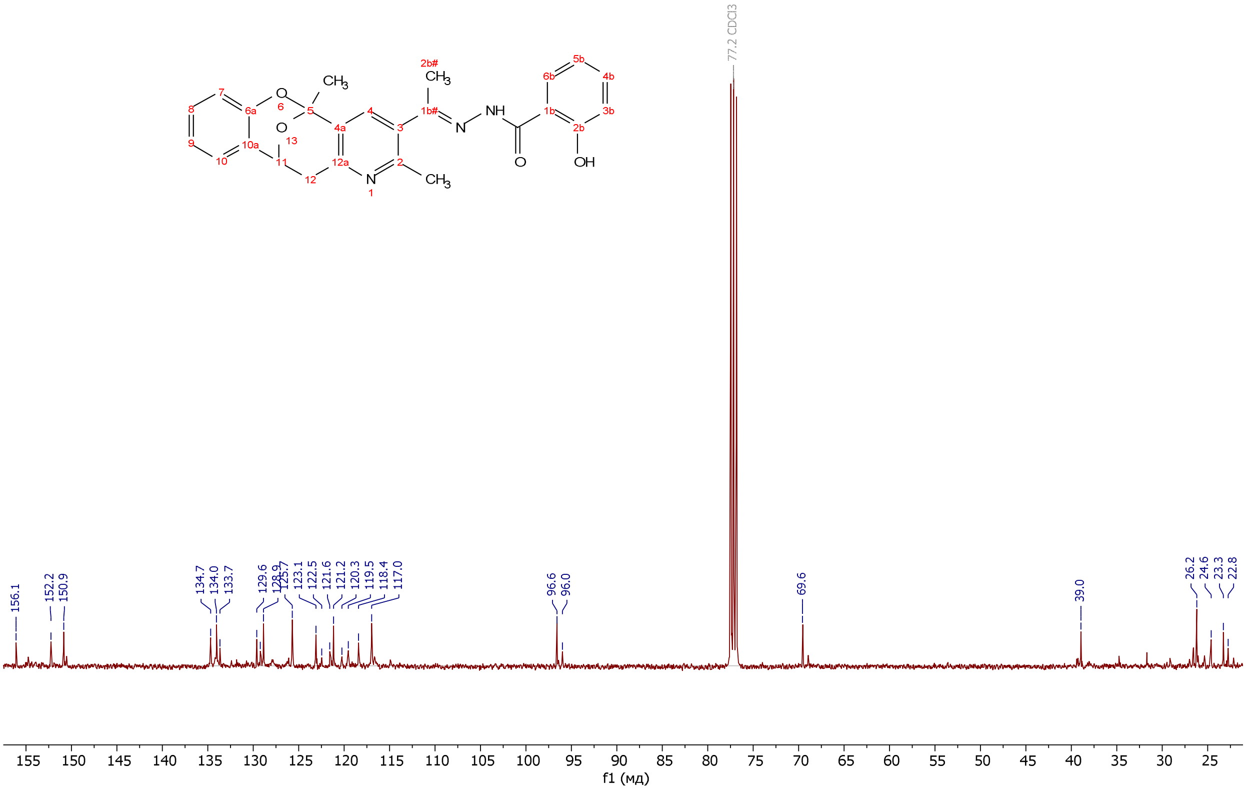

^{1}H (400 MHz, $CDCl_3$) and ^{13}C (100 MHz, $CDCl_3$) NMR Spectra of **4b**

	ppm	Hz	Hight
1	1.97	789.1	5.7
2	2.71	1082.1	4.7
3	3.19	1275.9	0.7
4	3.23	1292.7	0.8
5	3.68	1472.7	0.6
6	3.70	1478.8	0.6
7	3.73	1489.5	0.5
8	3.74	1495.6	0.5
9	5.41	2164.0	1.4
10	5.43	2170.1	1.2
11	6.74	2693.4	1.1
12	6.76	2702.6	1.3
13	6.84	2733.1	0.7

	ppm	Hz	Hight
1	6.88	2749.9	0.6
2	6.90	2757.5	1.1
3	6.92	2765.2	0.6
4	7.04	2815.5	1.2
5	7.06	2823.1	1.1
6	7.09	2833.8	0.8
7	7.11	2841.5	1.1
8	7.13	2849.1	0.5
9	8.07	3227.5	1.9

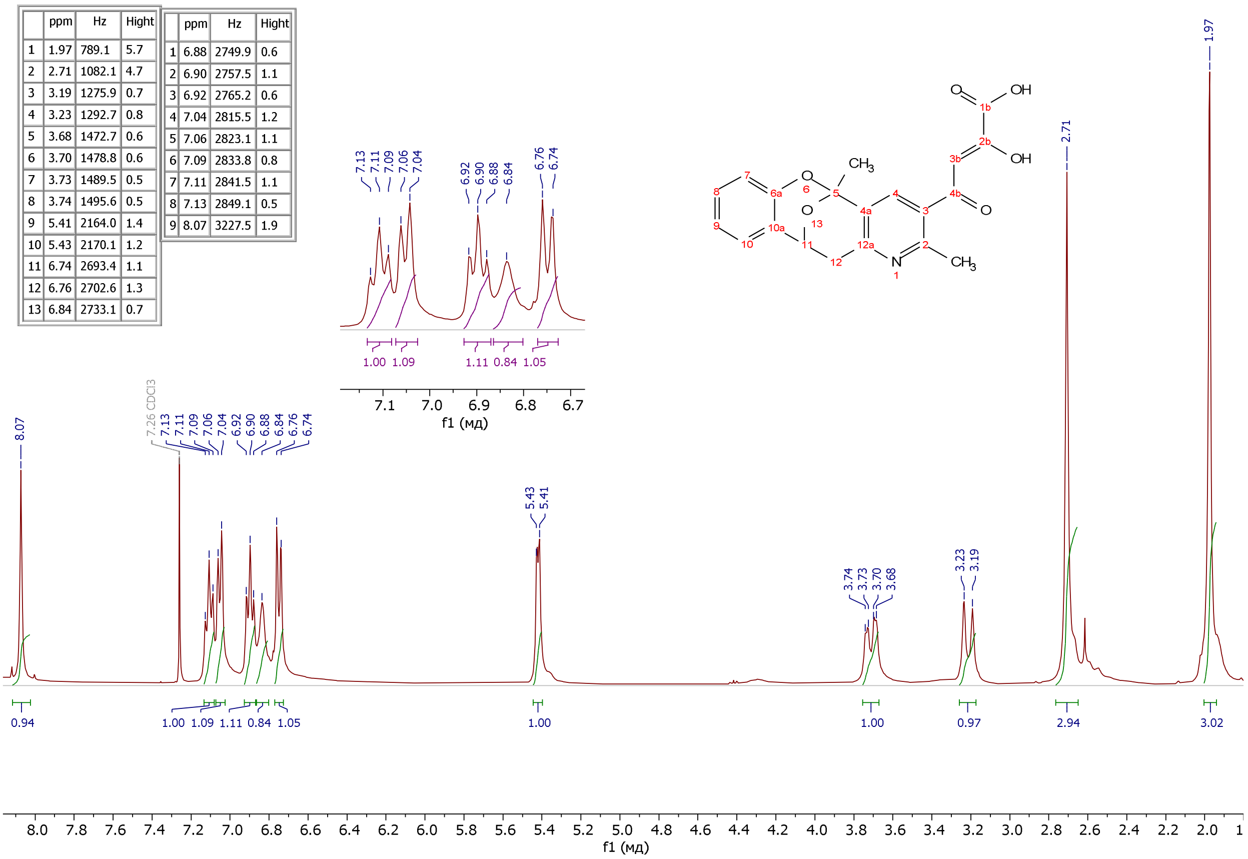

No.	(ppm)	(Hz)	Height	No.	(ppm)	(Hz)	Height	No.	(ppm)	(Hz)	Height
1	22.52	2263.8	0.0170	8	121.47	12210.7	0.0412	15	150.34	15113.0	0.0349
2	26.03	2616.5	0.0688	9	122.37	12300.8	0.0314	16	154.54	15534.7	0.0099
3	38.03	3823.2	0.0194	10	125.70	12636.3	0.0341	17	157.11	15793.5	0.0111
4	68.84	6920.0	0.0317	11	129.08	12975.6	0.0417	18	163.89	16475.0	0.0130
5	95.92	9642.0	0.0477	12	130.80	13149.1	0.0204	19	172.36	17326.1	0.0074
6	101.03	10155.8	0.0123	13	130.99	13168.2	0.0139	20	191.13	19213.3	0.0074
7	116.86	11747.8	0.0367	14	135.57	13628.3	0.0147				

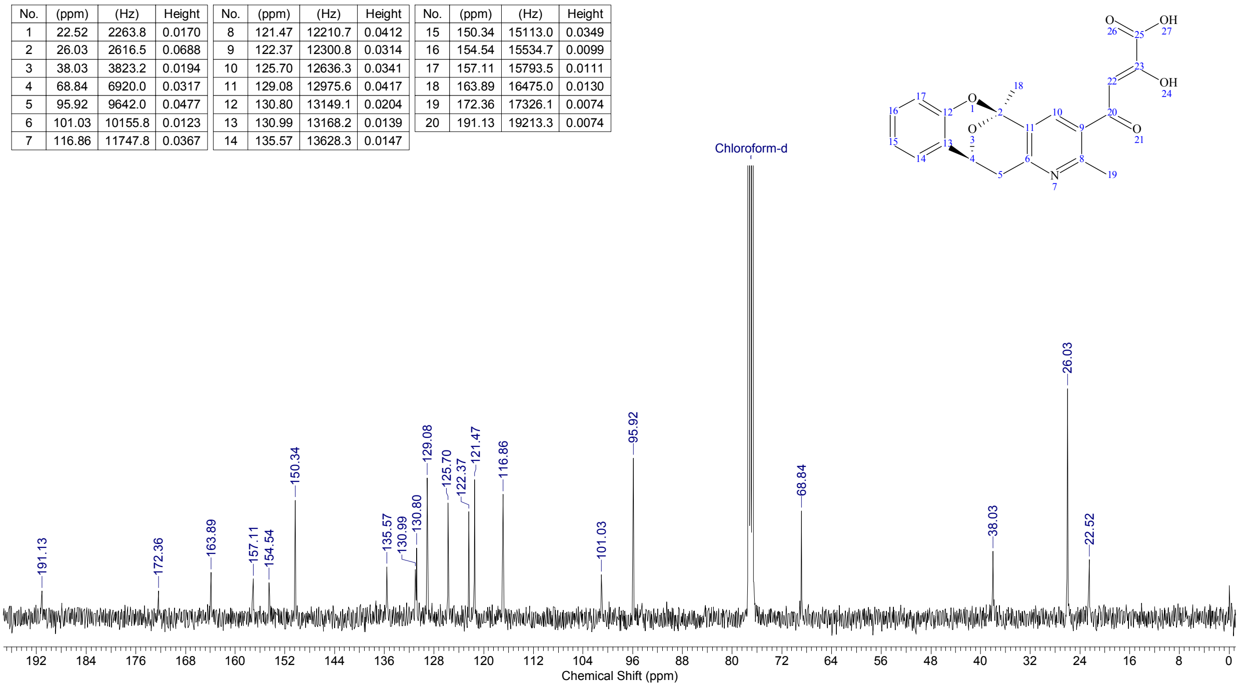

^{1}H (400 MHz, $CDCl_3$) and ^{13}C (100 MHz, $CDCl_3$) NMR Spectra of **5**

	ppm	Hz	Hight
1	1.95	781.6	5.1
2	2.65	1060.8	6.6
3	3.07	1228.6	1.0
4	3.12	1245.4	1.1
5	3.64	1456.0	0.6
6	3.66	1462.1	0.7
7	3.69	1474.3	0.6
8	3.70	1480.4	0.5
9	5.41	2162.5	1.3
10	5.42	2168.6	1.1
11	5.97	2386.8	1.7
12	6.70	2679.7	0.8
13	6.72	2687.4	0.9
14	6.87	2745.4	0.5

	ppm	Hz	Hight
1	6.89	2753.0	1.1
2	6.91	2760.6	0.7
3	7.05	2817.1	1.3
4	7.07	2824.7	1.0
5	7.08	2830.8	0.7
6	7.10	2836.9	1.1
7	7.12	2844.5	0.6
8	7.24	2893.4	0.6
9	7.25	2897.7	1.4
10	7.26	2902.5	3.7
11	7.28	2908.6	0.6
12	7.82	3125.3	1.7
13	8.36	3343.5	0.4
14	14.93	5968.0	0.9

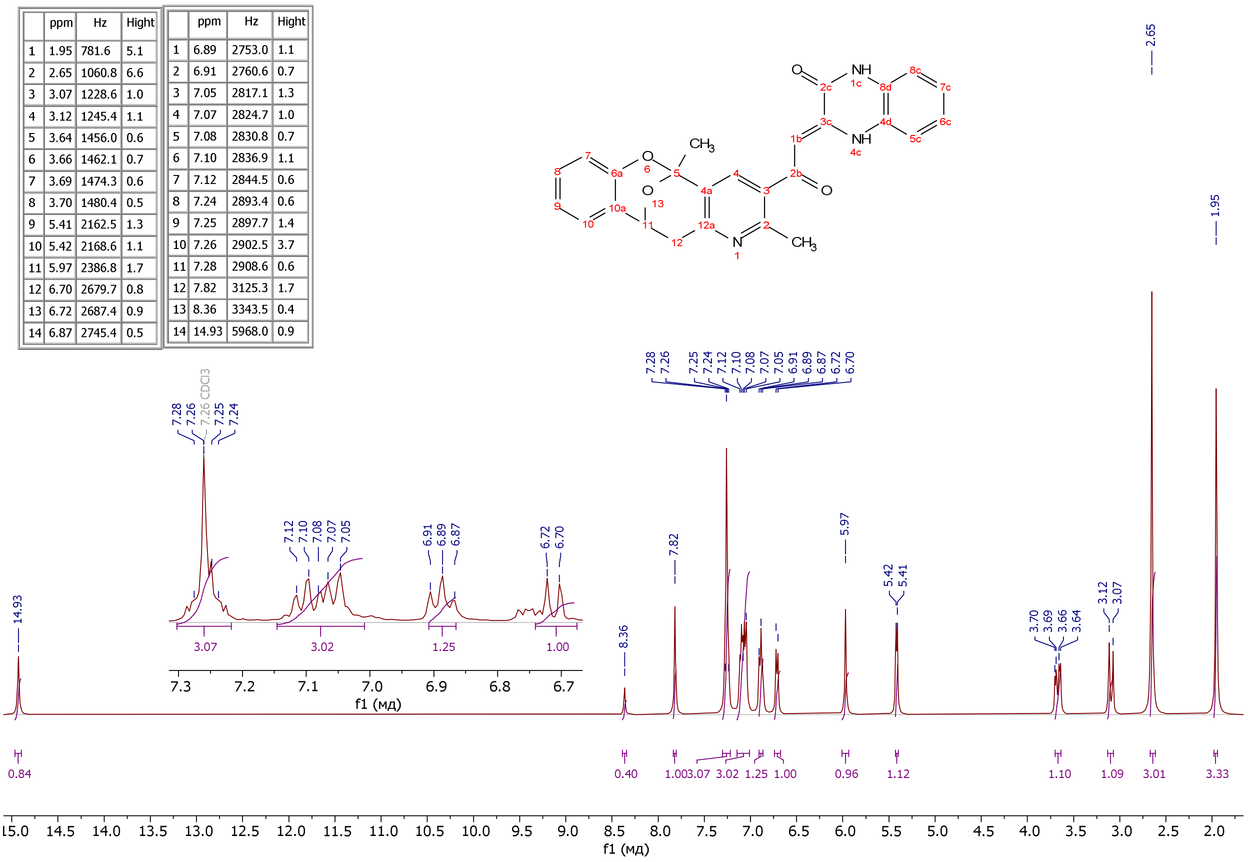

No.	(ppm)	(Hz)	Height	No.	(ppm)	(Hz)	Height	No.	(ppm)	(Hz)	Height
1	23.48	2360.6	0.0708	10	121.09	12172.4	0.0747	19	132.84	13354.2	0.0770
2	26.16	2629.9	0.0796	11	122.93	12357.4	0.0383	20	134.87	13557.4	0.0381
3	39.28	3948.8	0.0705	12	123.05	12369.8	0.0557	21	145.97	14674.0	0.0453
4	69.55	6991.9	0.0711	13	125.65	12630.5	0.0853	22	150.74	15153.2	0.0462
5	91.01	9148.4	0.0533	14	125.70	12636.3	0.0729	23	153.22	15402.4	0.0451
6	96.48	9698.6	0.0858	15	125.98	12664.1	0.0474	24	155.29	15610.4	0.0270
7	116.77	11738.2	0.0647	16	128.72	12939.2	0.0504	25	156.20	15702.4	0.0398
8	116.81	11742.0	0.0562	17	128.76	12944.0	0.0642	26	191.18	19218.1	0.0412
9	117.15	11776.6	0.0696	18	129.22	12990.0	0.0463				

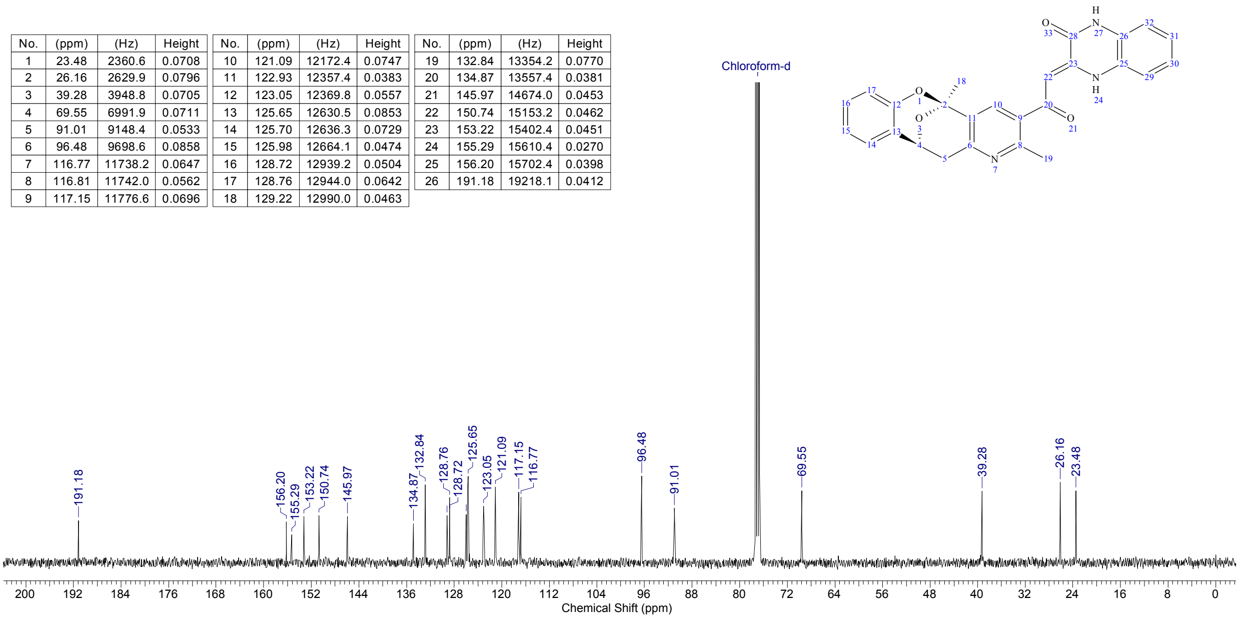

^{1}H (400 MHz, $CDCl_3$) and ^{13}C (100 MHz, $CDCl_3$) NMR Spectra of **6a**

	ppm	Hz	Hight
1	1.97	788.1	3.4
2	2.66	1062.0	3.7
3	3.05	1218.6	0.4
4	3.09	1236.0	0.5
5	3.63	1449.8	0.3
6	3.64	1455.3	0.3
7	3.67	1467.2	0.3
8	3.68	1472.7	0.3
9	5.40	2160.6	0.5
10	5.42	2166.1	0.5
11	6.68	2672.1	1.4
12	6.75	2700.5	0.5
13	6.78	2708.7	0.6
14	6.86	2744.5	0.2

	ppm	Hz	Hight
1	6.88	2751.8	0.5
2	6.90	2759.1	0.3
3	7.04	2814.1	0.5
4	7.06	2820.5	0.4
5	7.09	2834.7	0.2
6	7.11	2841.1	0.4
7	7.14	2853.5	0.6
8	7.15	2860.3	0.9
9	7.17	2868.1	0.1
10	7.20	2878.2	0.4
11	7.21	2883.7	1.0
12	7.24	2892.8	0.6
13	7.26	2902.5	0.4
14	7.91	3160.3	1.2

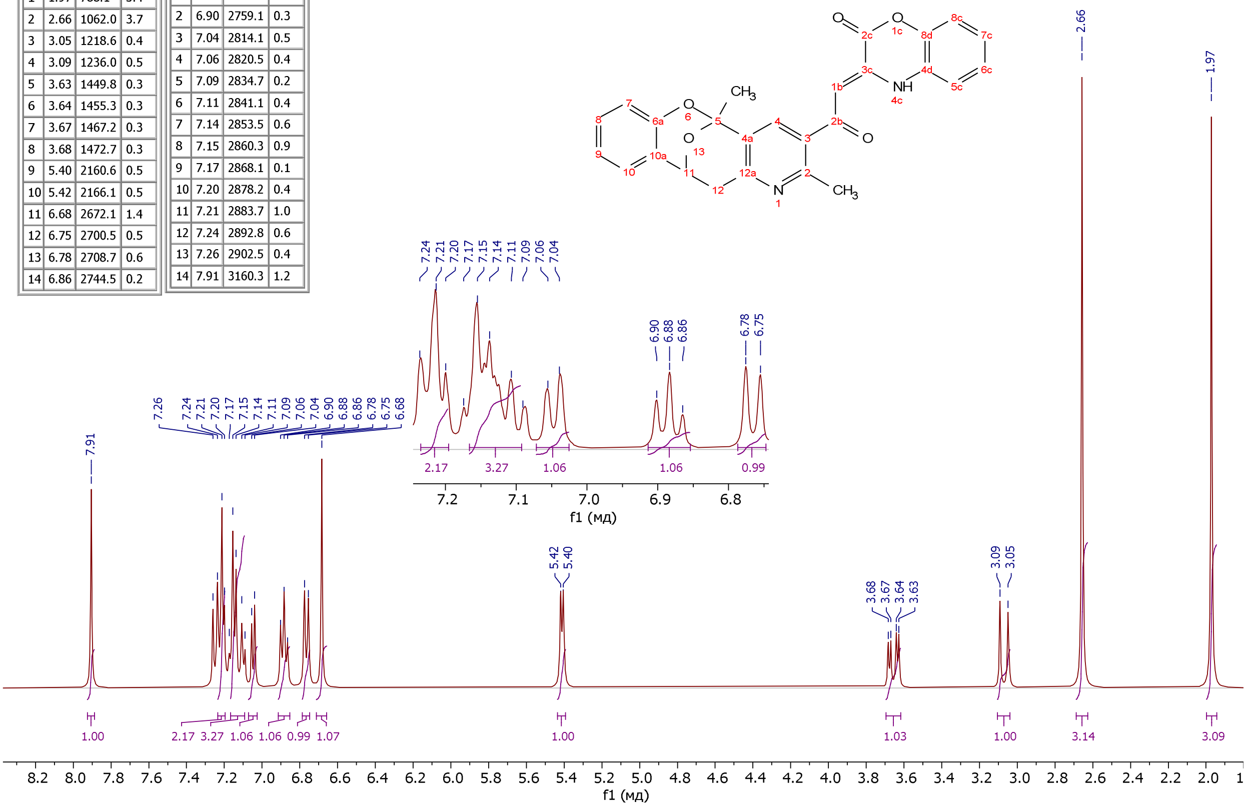

13C NMR (101 MHz, CHLOROFORM - *D*) δ 194.0, 157.3, 155.9, 154.3, 150.9, 141.5, 139.3, 133.7, 133.5, 129.5, 128.9, 126.1, 125.7, 124.6, 123.4, 123.1, 121.2, 117.3, 117.0, 116.3, 97.4, 96.6, 69.6, 39.5, 26.3, 23.9.

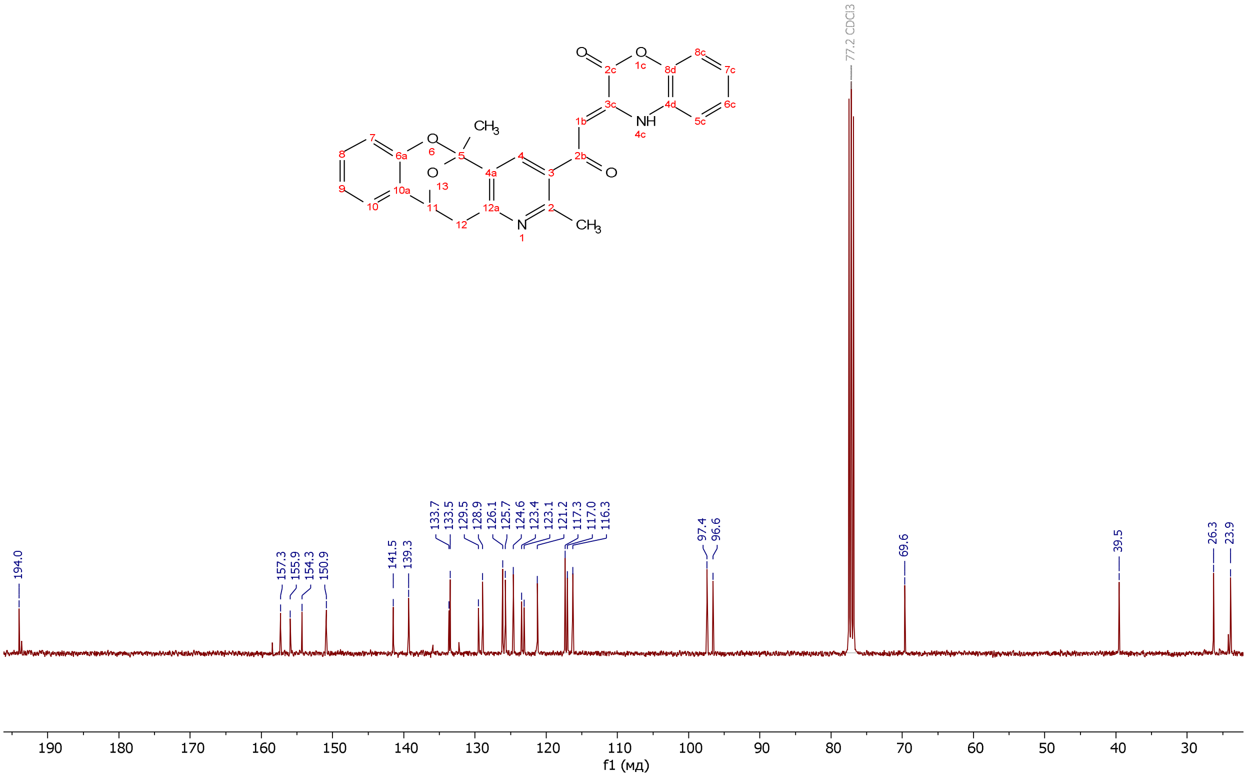

^{1}H (400 MHz, $CDCl_3$) and ^{13}C (100 MHz, $CDCl_3$) NMR Spectra of **6b**

	ppm	Hz	Hight
1	1.98	792.2	4.7
2	2.66	1065.3	4.7
3	3.07	1227.1	0.8
4	3.12	1245.4	0.8
5	3.64	1454.4	0.5
6	3.65	1460.5	0.5
7	3.68	1471.2	0.4
8	3.70	1477.3	0.4
9	5.41	2162.4	0.9
10	5.42	2168.5	0.9
11	6.76	2702.6	0.8
12	6.78	2710.2	0.9
13	6.86	2742.3	1.7
14	6.87	2748.4	0.6

	ppm	Hz	Hight
1	6.89	2756.0	0.9
2	6.91	2763.6	0.5
3	7.04	2814.0	1.0
4	7.06	2821.6	0.8
5	7.10	2838.4	0.5
6	7.12	2846.0	0.7
7	7.14	2853.7	0.3
8	7.23	2891.8	0.8
9	7.26	2902.5	1.2
10	7.96	3183.2	1.5
11	8.11	3241.2	1.4
12	8.13	3248.9	0.8
13	8.15	3258.0	0.7
14	12.90	5156.2	0.9

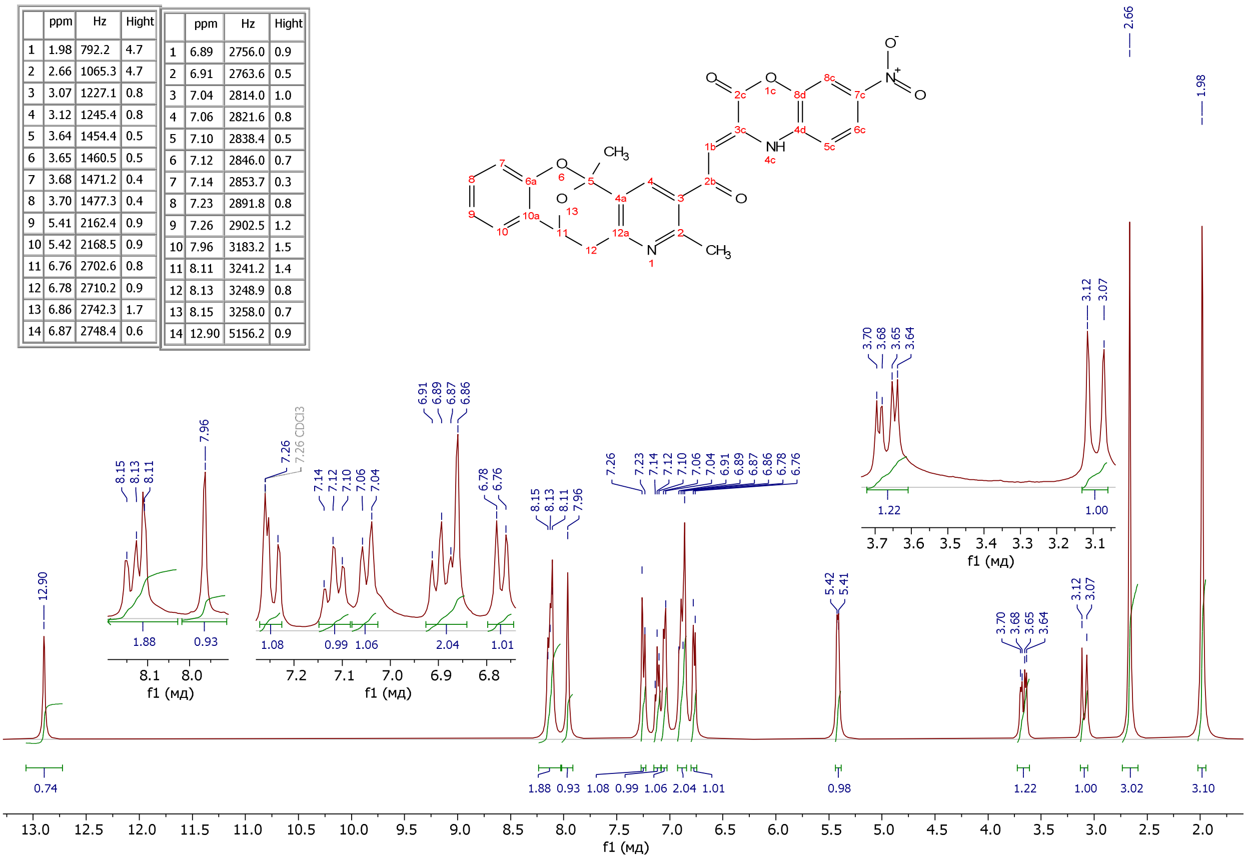

13C NMR (101 MHz, CHLOROFORM - *D*) δ 194.6, 157.7, 155.1, 154.7, 150.7, 143.2, 140.5, 137.5, 134.0, 133.0, 129.9, 129.1, 129.0, 125.8, 122.9, 121.9, 121.4, 117.0, 116.0, 113.6, 100.3, 96.4, 69.4, 39.4, 26.3, 23.8.

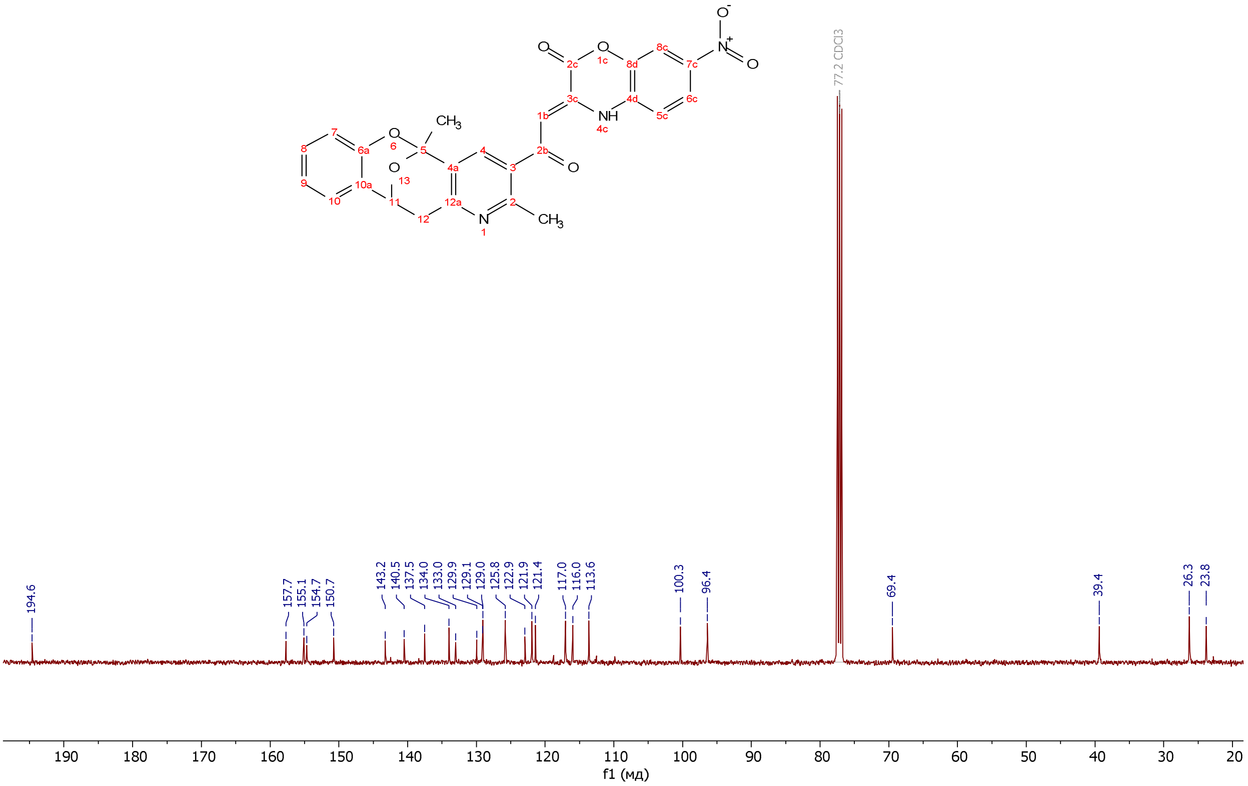

^{1}H (400 MHz, $CDCl_3$) and ^{13}C (100 MHz, $CDCl_3$) NMR Spectra of **6c**

	ppm	Hz	Hight
1	1.96	978.1	4428.9
2	2.55	1276.3	4695.6
3	2.68	1339.5	24.2
4	2.99	1497.1	509.8
5	3.03	1514.6	565.5
6	3.54	1771.4	336.8
7	3.55	1777.0	370.3
8	3.58	1788.9	323.1
9	3.59	1794.5	305.3
10	5.49	2745.7	584.9
11	5.50	2751.0	543.4
12	6.58	3291.6	2183.6
13	6.74	3371.0	463.5
14	6.74	3372.1	433.0
15	6.76	3379.1	508.3
16	6.76	3380.3	469.0
17	6.88	3442.0	218.9
18	6.88	3443.1	268.9
19	6.90	3449.5	562.1
20	6.90	3450.6	447.7
21	6.91	3457.0	327.0
22	6.91	3458.1	232.2

	ppm	Hz	Hight
23	7.09	3546.2	240.5
24	7.09	3547.9	271.9
25	7.11	3553.7	178.3
26	7.11	3556.0	142.9
27	7.12	3561.8	206.2
28	7.12	3563.4	190.5
29	7.21	3606.3	595.7
30	7.22	3611.1	1075.3
31	7.23	3614.4	568.2
32	7.24	3619.2	1091.0
33	7.72	3861.2	548.7
34	7.72	3862.6	517.8
35	7.74	3869.3	526.6
36	7.74	3870.7	484.8
37	8.08	4039.2	8.5
38	8.11	4054.2	1614.0
39	8.18	4091.7	599.3
40	8.18	4093.1	509.6
41	8.19	4096.5	590.2
42	8.19	4097.9	517.6
43	8.20	4103.1	13.6
44	12.59	6296.6	453.7

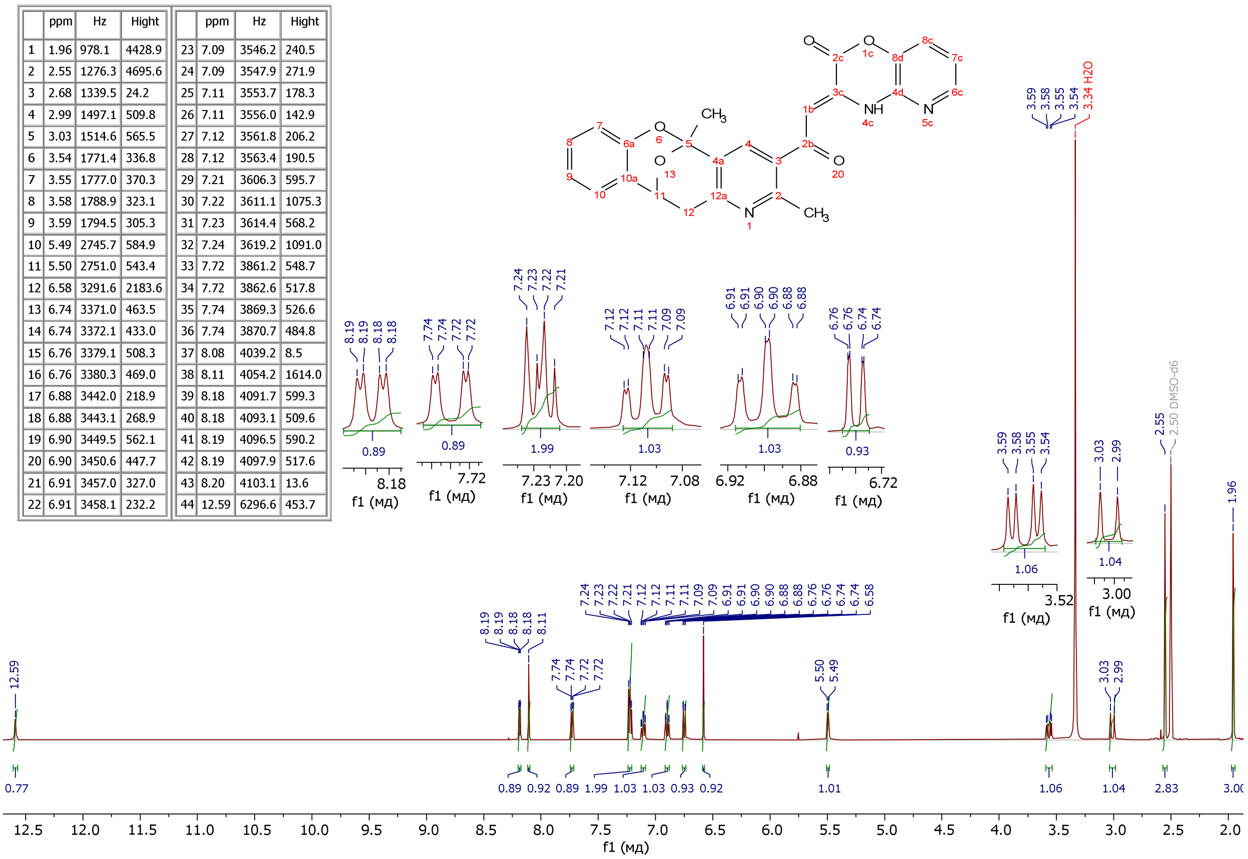

13C NMR (126 MHz, DMSO-d_6) δ 193.6, 156.3, 155.0, 154.0, 150.4, 144.3, 140.4, 137.5, 137.3, 133.4, 133.1, 129.0, 128.7, 126.1, 124.1, 123.4, 121.0, 119.8, 116.1, 97.4, 96.4, 68.6, 38.9, 25.7, 23.3.

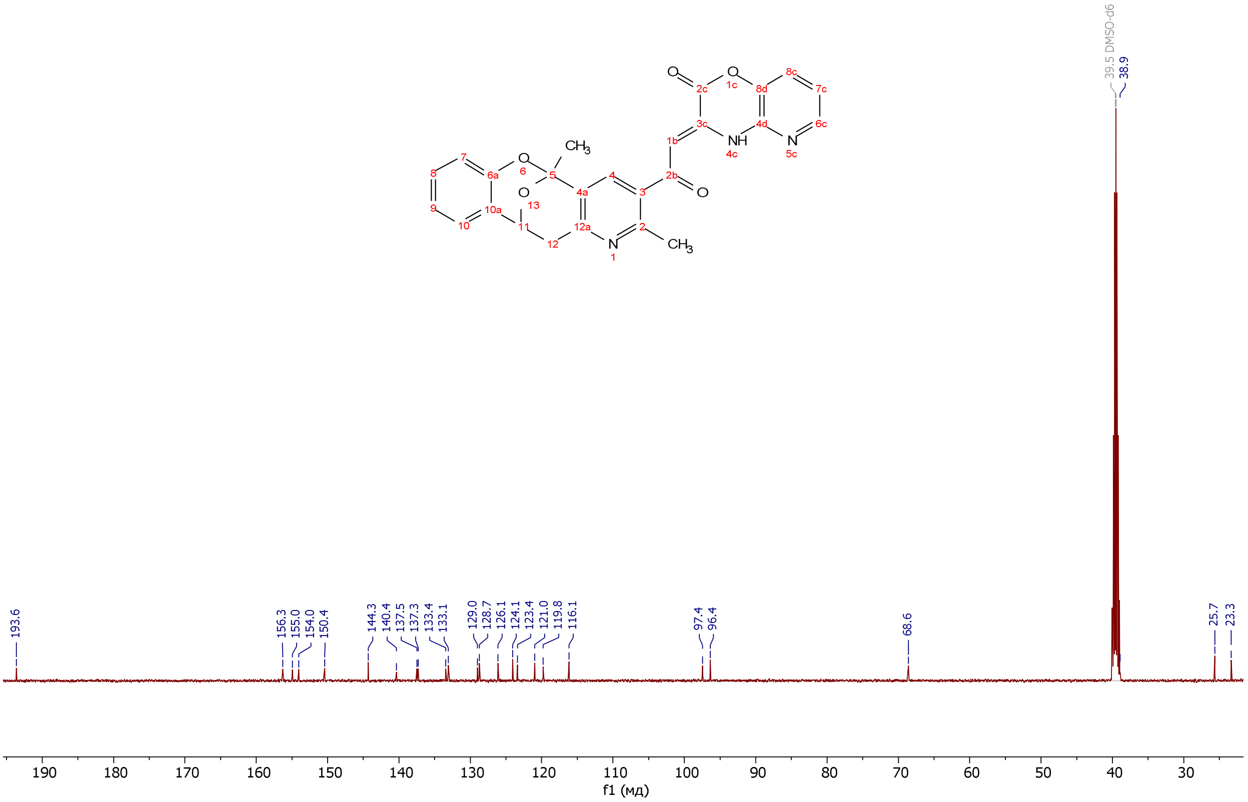

^{1}H (500 MHz, DMSO-d_6) and ^{13}C (125 MHz, DMSO-d_6) NMR Spectra of **6d**

Copies of MS Spectra of Products

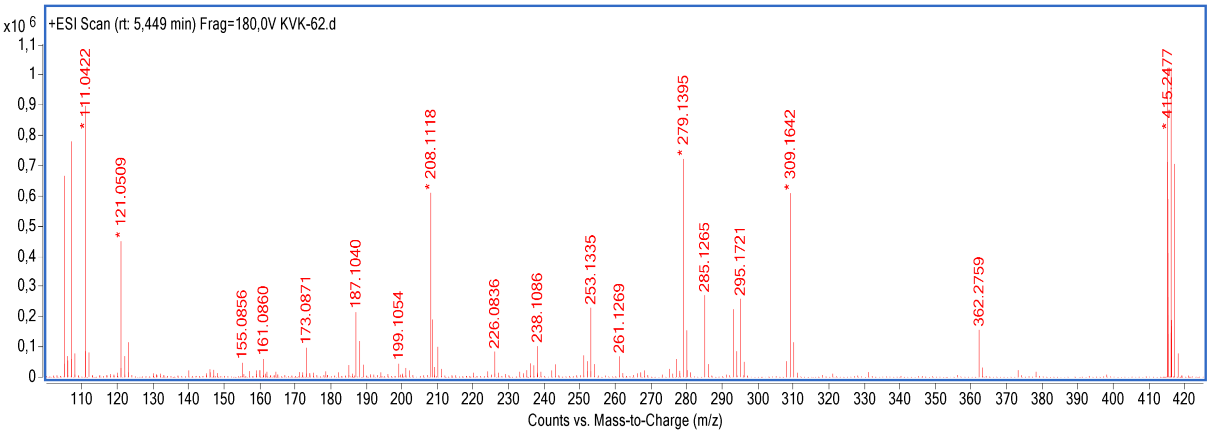

Mass spectrum (LC/Q-TOF) of **4a** (t_R = 5,499 min)

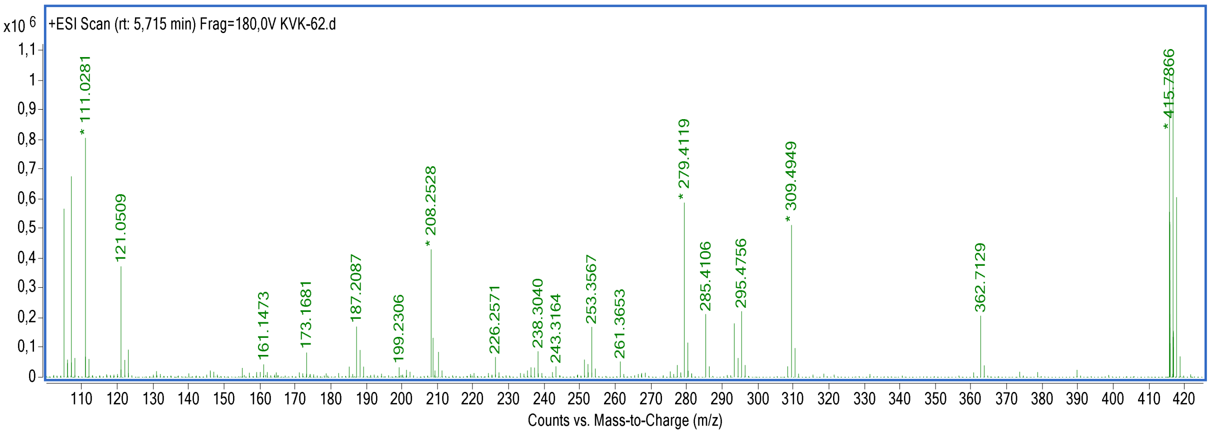

Mass spectrum (LC/Q-TOF) of **4a** (t_R = 5,715 min)

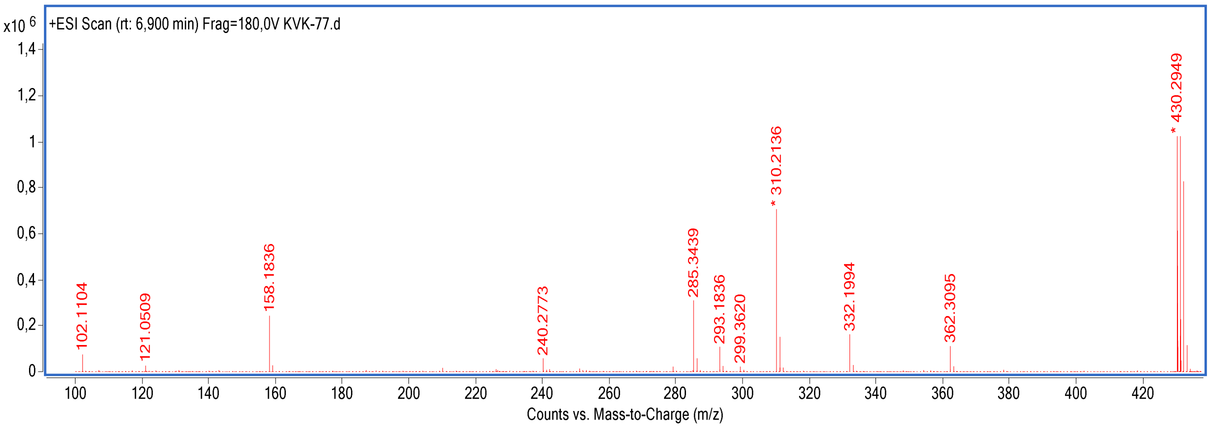

Mass spectrum (LC/Q-TOF) of **4b**

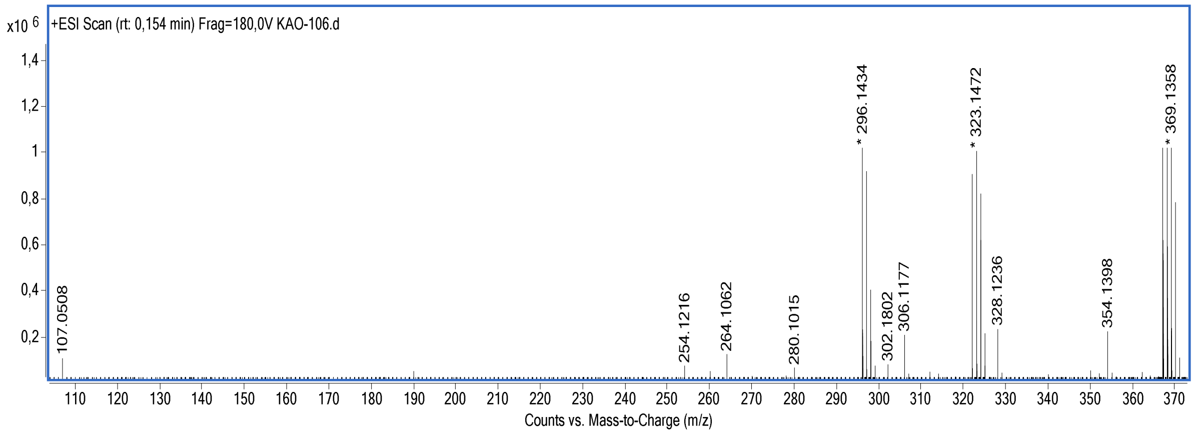

Mass spectrum (LC/Q-TOF) of **5**

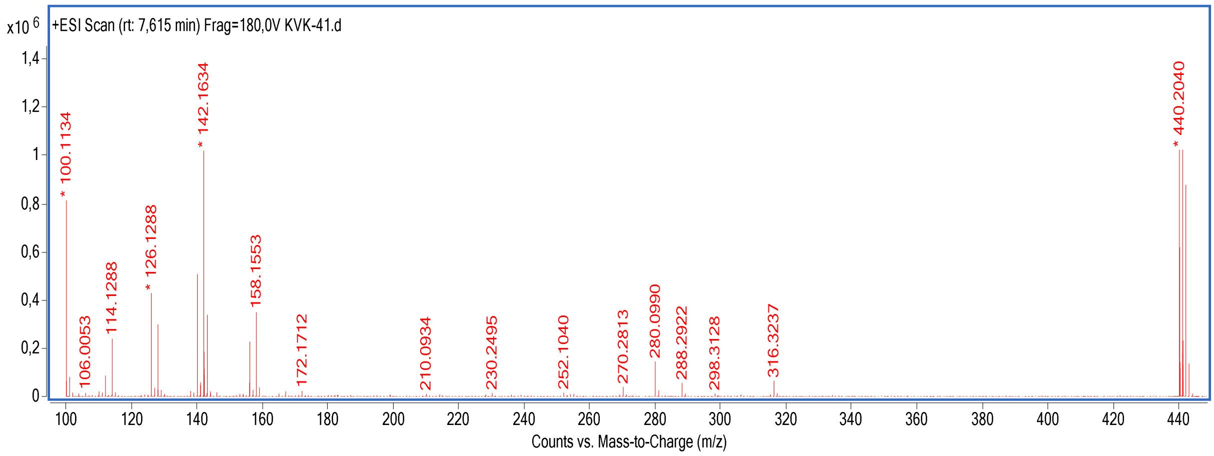

Mass spectrum (LC/Q-TOF) of **6a**

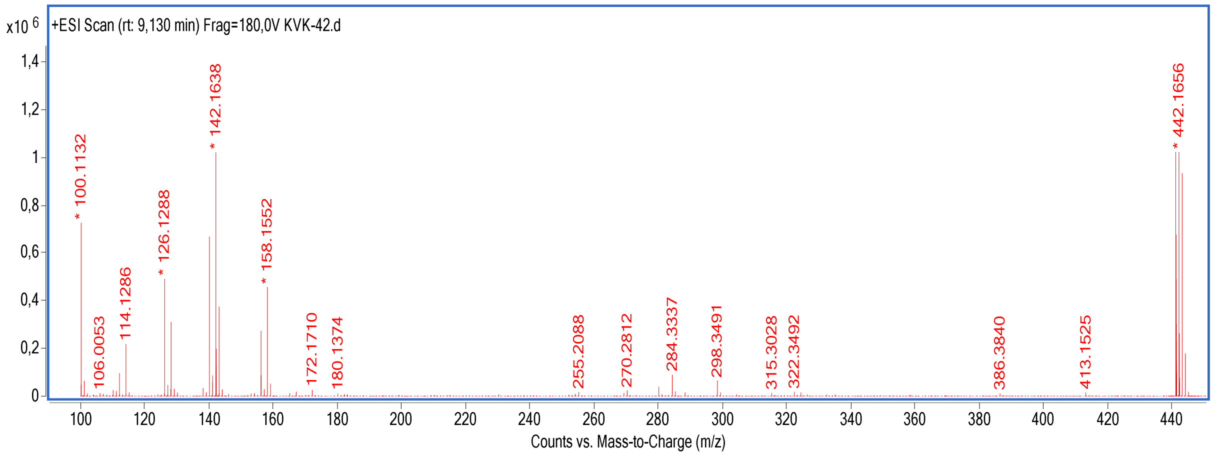

Mass spectrum (LC/Q-TOF) of **6b**

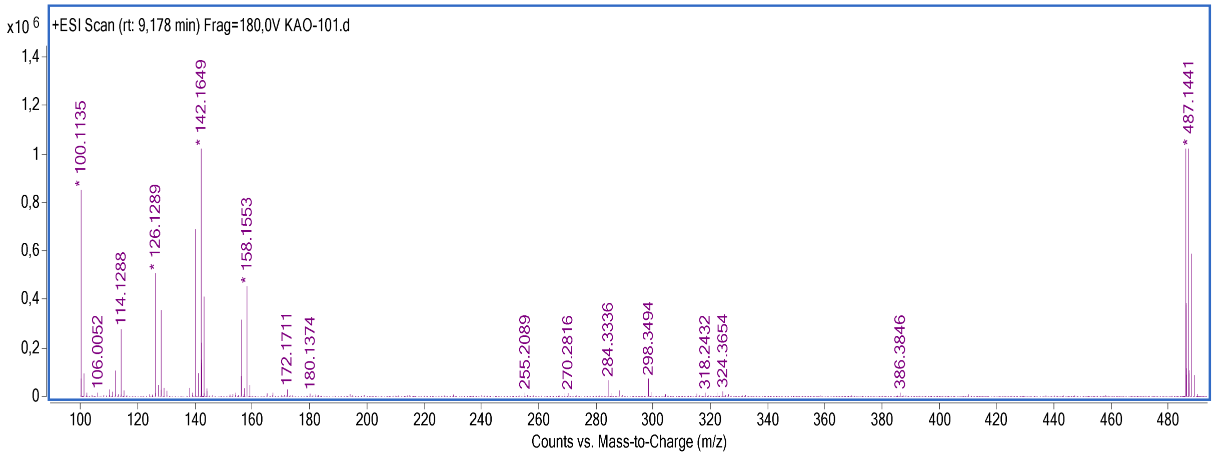

Mass spectrum (LC/Q-TOF) of **6c**

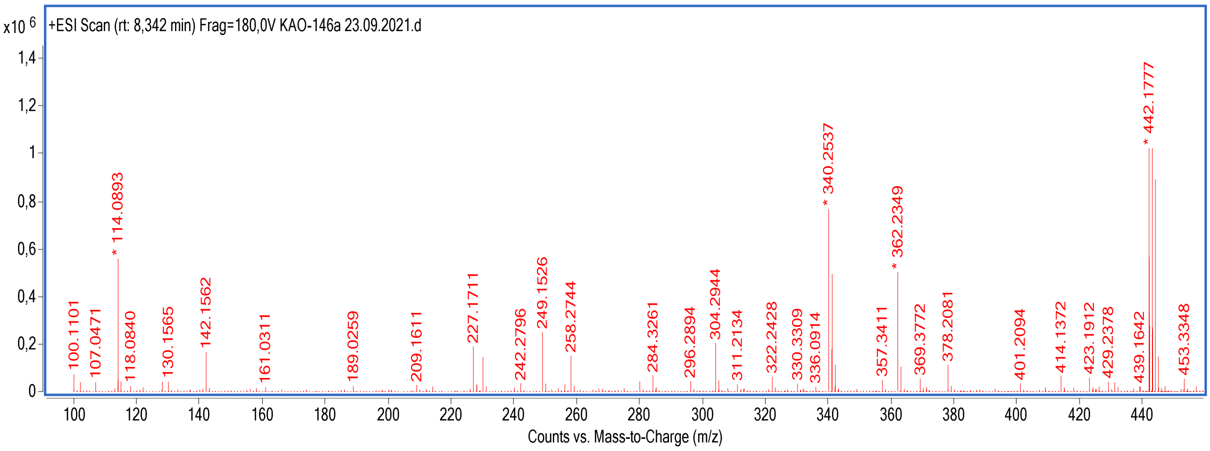

Mass spectrum (LC/Q-TOF) of **6d**

Biological tests

Protocol 1

Measuring 50% cytotoxic (inhibitory) concentration IC50

Note: the used method is like virus titering using Reed-Muench method, the difference is that substances are added with a virus.

Note: tested concentration range: 0.091 – 200 ug/ml;

Note: total duration of the experiment is 3-5 days.

Needed components:

Maintenance medium: growth medium with all additives, with 10% fetal bovine serum (FBS).

Infection medium: growth medium with all additives, but with 1% heat-inactivated (HI) FBS.

1) **Preparation of tested substances**

Weight 10 mg of a substance and dissolve it in 0.5 ml of DMSO. By our experience, DMSO has the best ability to dissolve compounds if compared to water or alcohols.

Note: This solution (20 mg/ml) will be then diluted 100 times to prepare a sample with the highest concentration (200 ug/ml), row-H sample. Note: the DMSO concentration in samples added to cells should not exceed 1%. The row-H sample is the highest effective concentration of a substance (200 ug/ml).

Note: Row H requires 150 µl *(12) wells = 1.8 ml of the row-H sample.

2) **To prepare the row-H sample, aliquot 1782 µl of infection medium, add 18 µl of the DMSO solution of a substance (20 mg/ml).**

3) **Seeding 96-well plate.**

Vero E6 cells are routinely maintained in Maintenance medium with 10% serum.

3.1. Grow a sufficient number of Vero E6 cells. One 96-well plate requires 96 * 100 µl * 2.0 x 10^5 cells = 1.9 million cells.

One confluent dish P150 is sufficient to seed 6 plates.

3.2. Detach cells with trypsin and dilute in 20 ml of Infection medium with 1% FBS. (Note: at this step, you need Infection medium with 1% FBS, and not the standard medium). Break up cell clumps by pipetting. Determine the cell counts using a hemocytometer. Dilute the cell

suspension to 2.0 x 10^5 cells/mL (200,000 cells/mL). Determine the cell counts again using a hemocytometer to exclude errors.

3.3. Dispense 100 µl (of 2.0 x 10^5 cells/ml) cell suspension (20,000 cells per well) into all wells in a 96-well plate.

4) IC50 Test procedure

Note: Infection medium with 1% HI FBS is used for all dilutions after this step.

Note: All substances are added in rows 1-10. Substances are not added to rows 11 and 12. Row 12 is control of normal cell growth in the absence of a substance and a virus. Row 11 is control of infection in the absence of a substance.

Wait until Vero E6 cells are fully attached, then continue with the protocol.

4.1. Remove media from all wells.

4.2. In rows A-G (rows 1 to 10), add 100 µl of Infection medium. In all wells of rows 11 and 12, add 100 µl of Infection medium.

The scheme of the resulting plate at this stage: Yellow indicates wells in which 100 ul of Infection medium was added.

	1	2	3	4	5	6	7	8	9	10	11	12
A												
B												
C												
D												
E												
F												
G												
H												

4.3. In row H, in wells 1-10, add 150 µl of the row-H sample (200 ug/ml substance in Infection medium).

4.4. Next, transfer 50 µl from the previous row to the next row (from H to G, from G to F, and so on). Mix each time by pipetting.

The scheme of the resulting plate at this stage:

	1	2	3	4	5	6	7	8	9	10	11	12
A	0,091	0,091	0,091	0,091	0,091	0,091	0,091	0,091	0,091	0,091	0	0
B	0,27	0,27	0,27	0,27	0,27	0,27	0,27	0,27	0,27	0,27	0	0
C	0,82	0,82	0,82	0,82	0,82	0,82	0,82	0,82	0,82	0,82	0	0
D	2,47	2,47	2,47	2,47	2,47	2,47	2,47	2,47	2,47	2,47	0	0
E	7,4	7,4	7,4	7,4	7,4	7,4	7,4	7,4	7,4	7,4	0	0
F	22,2	22,2	22,2	22,2	22,2	22,2	22,2	22,2	22,2	22,2	0	0
G	66,7	66,7	66,7	66,7	66,7	66,7	66,7	66,7	66,7	66,7	0	0
H	200	200	200	200	200	200	200	200	200	200	0	0

Yellow - Infection medium without substances;

Green - Infection medium with substances. Numbers are concentrations of the substance (ug/ml).

5) The test continued

Incubate the plate in CO2-incubator for 3 days. Control development of CPE at day 3.

6) Stopping the incubation and making measurements:

6.1. Add 20 µl of MTT solution (3 mg/ml) in 1X MEM (no additives) to all wells. Do not remove the previous medium from the wells. Incubate the plate with MTT for 2-3 hours in a CO2-incubator.

6.2. Check that coloration of cell monolayers fully developed. Gently remove the medium from all wells, taking care not to disturb cells in the monolayers (may be attached loosely). There is no need to wash the wells of the plate. Just quantitatively remove the medium. Add 100 µl of DMSO (with addition of 1% glacial acetic acid) to all wells.

6.3. Dissolve the formazan and read the plate at a wavelength 595 nm.

Notes:

1. Effective concentrations of substances in rows A-H

	Rows 1-10, ug/ml	Rows 11-12
A	0.091	0
B	0.27	0

C	0.82	0
D	2.5	0
E	7.4	0
F	22.2	0
G	66.7	0
H	200	0

Protocol 2.

Measuring antiviral 50% efficient concentration (EC50) using Cytopathic Endpoint Assay (CEA)

Note: the used method CEA is like virus titering using Reed-Muench method, the difference is that substances are added with a virus.

Note: tested concentration range: 0.091 – 200 ug/ml;

Note: we use multiplicity of infection: 2000 PFU per well;

Note: total duration of the experiment is 3-5 days.

Needed components:

<u>Maintenance medium:</u> growth medium with all additives, with 10% fetal bovine serum (FBS).

<u>Infection medium:</u> growth medium with all additives, but with 1% heat-inactivated (HI) FBS.

6) **Preparation of tested substances**

Weight 10 mg of a substance and dissolve it in 0.5 ml of DMSO. By our experience, DMSO has the best ability to dissolve compounds if compared to water or alcohols.

Note: This solution (20 mg/ml) will be then diluted 100 times to prepare a sample with the highest concentration (200 ug/ml), row-H sample. Note: the DMSO concentration in samples added to cells should not exceed 1%. The row-H sample is the highest effective concentration of a substance (200 ug/ml).

Note: Row H requires 150 µl *(12) wells = 1.8 ml of the row-H sample.

7) **To prepare the row-H sample, aliquot 1782 µl of infection medium, add 18 µl of the DMSO solution of a substance (20 mg/ml).**

8) **Seeding 96-well plate.**

Vero E6 cells are routinely maintained in Maintenance medium with 10% serum.

3.1. Grow a sufficient number of Vero E6 cells. One 96-well plate requires 96 * 100 µl * 2.0 x 10^5 cells = 1.9 million cells.

One confluent dish P150 is sufficient to seed 6 plates.

3.2. Detach cells with trypsin and dilute in 20 ml of Infection medium with 1% FBS. (Note: at this step, you need Infection medium with 1% FBS, and not the standard medium). Break up cell clumps by pipetting. Determine the cell counts using a hemocytometer. Dilute the cell suspension to 2.0 x 10^5 cells/mL (200,000 cells/mL). Determine the cell counts again using a hemocytometer to exclude errors.

3.3. Dispense 100 µl (of 2.0 x 10^5 cells/ml) cell suspension (20,000 cells per well) into all wells in a 96-well plate.

9) CEA Test procedure

Note: Infection medium with 1% HI FBS is used for all dilutions after this step.

Note: All substances are added in rows 1-10. Substances are not added to rows 11 and 12. Row 12 is control of normal cell growth in the absence of a substance and a virus. Row 11 is control of infection in the absence of a substance.

Wait until Vero E6 cells are fully attached, then continue with the protocol.

4.1. Remove media from all wells.

4.2. In rows A-G (rows 1 to 10), add 100 µl of Infection medium. In all wells of rows 11 and 12, add 100 µl of Infection medium.

The scheme of the resulting plate at this stage: Yellow indicates wells in which 100 ul of Infection medium was added.

	1	2	3	4	5	6	7	8	9	10	11	12
A												
B												
C												
D												
E												
F												
G												
H												

4.3. In row H, in wells 1-10, add 150 µl of the row-H sample (200 ug/ml substance in Infection medium).

4.4. Next, transfer 50 µl from the previous row to the next row (from H to G, from G to F, and so on). Mix each time by pipetting.

4.5. When all plates are ready, add 20 µl of virus (1000 PFU per well) to the wells of rows 1-11. Note: row 12 remains uninfected.

It is convenient to add the virus using 8-channel pipettor. The virus stock titer should be adjusted to give a dose of 2000 PFU per well (i.e. 2x10^5 PFU/ml).

The scheme of the resulting plate at this stage:

	1	2	3	4	5	6	7	8	9	10	11	12
A	0,091	0,091	0,091	0,091	0,091	0,091	0,091	0,091	0,091	0,091	0	0
B	0,27	0,27	0,27	0,27	0,27	0,27	0,27	0,27	0,27	0,27	0	0
C	0,82	0,82	0,82	0,82	0,82	0,82	0,82	0,82	0,82	0,82	0	0
D	2,47	2,47	2,47	2,47	2,47	2,47	2,47	2,47	2,47	2,47	0	0
E	7,4	7,4	7,4	7,4	7,4	7,4	7,4	7,4	7,4	7,4	0	0
F	22,2	22,2	22,2	22,2	22,2	22,2	22,2	22,2	22,2	22,2	0	0
G	66,7	66,7	66,7	66,7	66,7	66,7	66,7	66,7	66,7	66,7	0	0
H	200	200	200	200	200	200	200	200	200	200	0	0

Yellow - Infection medium without substances and virus;

Green - Infection medium with a virus, without substances;

Red - Infection medium with a substance and with the virus. Numbers are concentrations of the substance (ug/ml).

10) The test continued

Incubate the plate in CO2-incubator for 3 days. Control development of CPE at day 3.

6) Stopping the incubation and making measurements:

6.1. Add 20 µl of MTT solution (3 mg/ml) in 1X MEM (no additives) to all wells. Do not remove the previous medium from the wells. Incubate the plate with MTT for 2-3 hours in a CO2-incubator.

6.2. Check that coloration of cell monolayers fully developed. Gently remove the medium from all wells, taking care not to disturb cells in the monolayers (may be attached loosely). There is no

need to wash the wells of the plate. Just quantitatively remove the medium. Add 100 µl of DMSO (with addition of 1% glacial acetic acid) to all wells.

6.3. Dissolve the formazan and read the plate at a wavelength 595 nm.

Notes:

2. Effective concentrations of substances in rows A-H

	Rows 1-10, ug/ml	Rows 11-12
A	0.091	0
B	0.27	0
C	0.82	0
D	2.5	0
E	7.4	0
F	22.2	0
G	66.7	0
H	200	0

3. This protocol was successfully used with SARS-CoV-2 virus.

Example of calculating a virus dilution to obtain 2000 PFU/well.

To reduce pipetting errors, we use the addition of a minimum 20 ul of a virus. Then the required titer is 100,000 PFU/ml – if 10 ul of this preparation is used this is 2000 PFU.

Out SARS-CoV-2 virus stock is 3.01E+06 PFU/ml.

Then we take 33 µl the virus stock to add to 966 µl of Infection medium

The resulting titer is 33 * 3,000,000/1000 = 99,000 PFU/ml

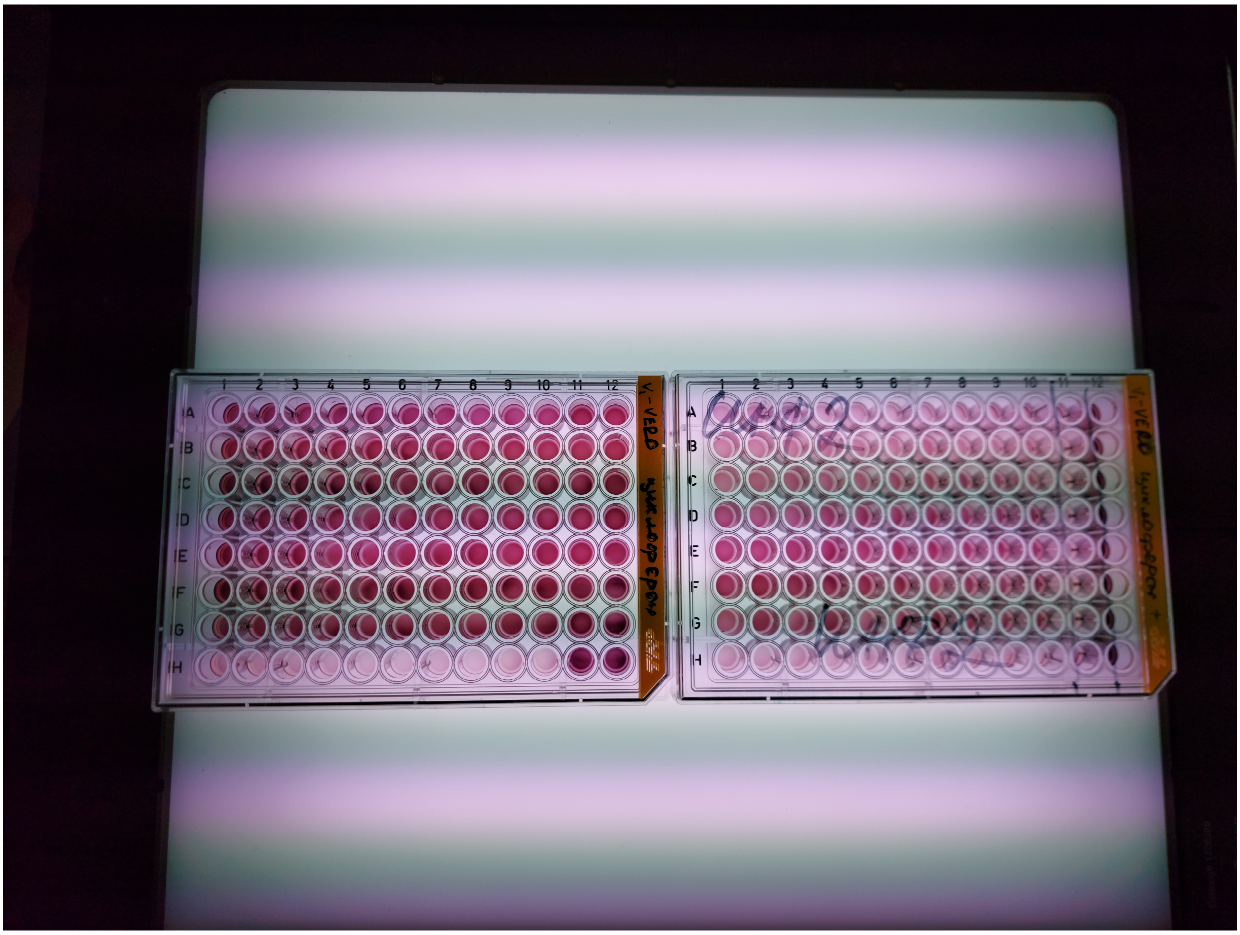

Presented is a photograph of two 96-well plates from an experiment to determine IC50 and EC50 for the control substance Cycloferon (Cridanimod) which is active against SARS-CoV-2.

One plate (left) was used to measure own cytotoxicity (IC50) of Cycloferon. The other plate (right) was used to measure the antiviral effect (EC50). With this example, simple visual inspection of the left plate already convince that the tested substance is not cytotoxic at concentrations 66.7 ug/ml or less. Also, the appearance of the right plate (note rows D,E,F,G) is indicative of virus-inhibiting activity in a concentration range 2.5-66.7 ug/ml. Rows D-G (right plate) show cells growth comparable to uninfected controls. Row H (right plate) is deficient in cells due to the cytotoxicity. Rows A,B,C (right plate) have fewer cells than D-G because the drug concentration is insufficient to inhibit SARS-CoV-2 and the virus kills the cells.

Vertical rows 1-10 are for the tested substance. Rows 11 and 12 are controls as described in the presented Protocols.

Figure - Examples of implementation of the protocols for measuring IC50 and EC50.
